# Tea polyphenol-engineered hybrid cellular nanovesicles for cancer immunotherapy and androgen deprivation therapy

**DOI:** 10.1186/s12951-024-02458-9

**Published:** 2024-04-18

**Authors:** Yiming Guo, Jicheng Wu, Lefan Chen, Lujie Liu, Tianxiang Bi, Yuanwei Pan, Qian-Fang Meng, Chaoliang Wang, Lang Rao, Qi Li

**Affiliations:** 1 Department of Urology, The First Affiliated Hospital of Zhengzhou University, Zhengzhou, Henan 450052 China; 2 Institute of Biomedical Health Technology and Engineering, Shenzhen Bay Laboratory, Shenzhen, Guangdong 518132 China; 3 Cancer Center, Renmin Hospital of Wuhan University, Wuhan, Hubei 430060 China

**Keywords:** Prostate cancer, Androgen deprivation therapy, Immunotherapy, Nanotechnology, Biomaterials

## Abstract

Androgen deprivation therapy (ADT) is a crucial and effective strategy for prostate cancer, while systemic administration may cause profound side effects on normal tissues. More importantly, the ADT can easily lead to resistance by involving the activation of NF-κB signaling pathway and high infiltration of M2 macrophages in tumor microenvironment (TME). Herein, we developed a biomimetic nanotherapeutic platform by deriving cell membrane nanovesicles from cancer cells and probiotics to yield the hybrid cellular nanovesicles (hNVs), loading flutamide (Flu) into the resulting hNVs, and finally modifying the hNVs@Flu with Epigallocatechin-3-gallate (EGCG). In this nanotherapeutic platform, the hNVs significantly improved the accumulation of hNVs@Flu-EGCG in tumor sites and reprogramed immunosuppressive M2 macrophages into antitumorigenic M1 macrophages, the Flu acted on androgen receptors and inhibited tumor proliferation, and the EGCG promoted apoptosis of prostate cancer cells by inhibiting the NF-κB pathway, thus synergistically stimulating the antitumor immunity and reducing the side effects and resistance of ADT. In a prostate cancer mouse model, the hNVs@Flu-EGCG significantly extended the lifespan of mice with tumors and led to an 81.78% reduction in tumor growth compared with the untreated group. Overall, the hNVs@Flu-EGCG are safe, modifiable, and effective, thus offering a promising platform for effective therapeutics of prostate cancer.

**Graphical Abstract:**

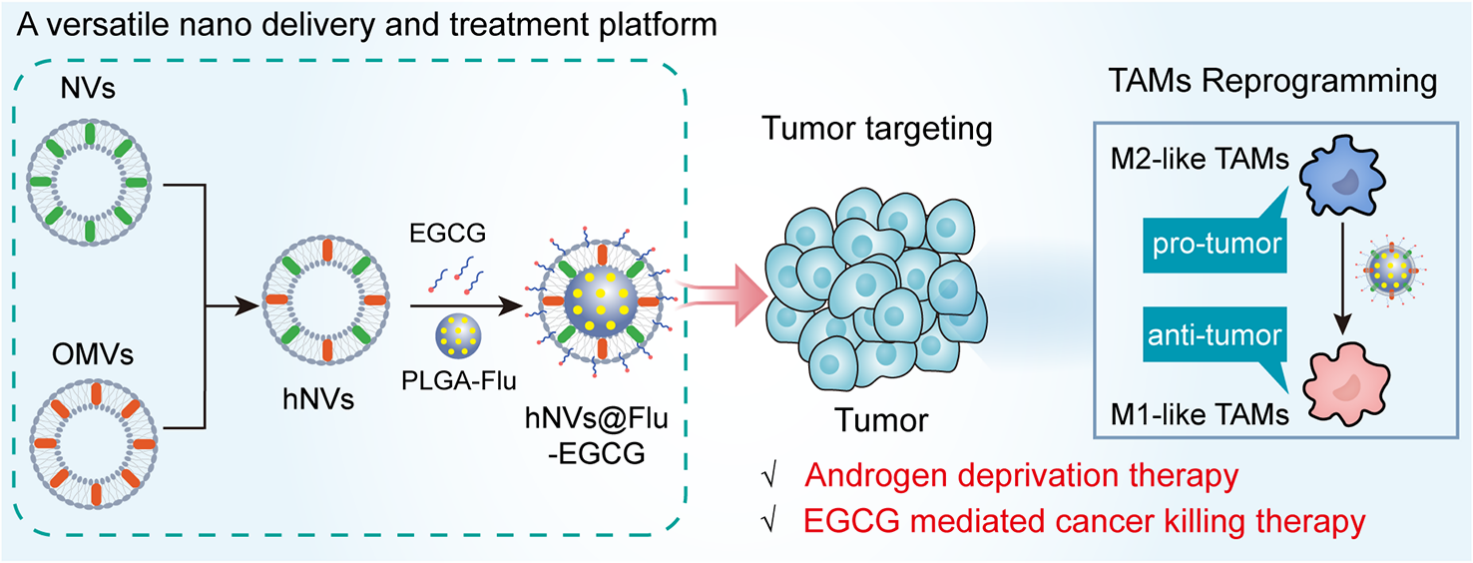

**Supplementary Information:**

The online version contains supplementary material available at 10.1186/s12951-024-02458-9.

## Introduction

As the second most commonly occurring cancer in men, prostate cancer ranks among the top five leading causes of cancer-related fatalities worldwide [1]. Since early-stage prostate cancer often presents with insidious and mild symptoms, most male patients are diagnosed at an advanced stage [2]. Although early-stage prostate cancer confined to the prostate tissue can be effectively treated through radical surgical resection, the majority of patients may still experience local recurrence and distant metastasis postoperatively, leading to a 5-year survival rate of just 14-34% [3]. Prostate cancer is a disease that relies on androgens for growth. The androgen receptor is expressed in almost all prostate cancer cells and the progression of this disease is largely dependent on androgen/androgen receptor signaling, which means inhibiting the production of androgens or antagonizing the effects of androgen receptors in the body through endocrine therapy can achieve the purpose of treating prostate cancer [4]. ADT remains the mainstream treatment for prostate cancer worldwide [5]. The aim of ADT is to hinder the onset and progression of prostate cancer by lowering the level of androgens in the body, with the goal of extending the patient’s survival and enhancing their quality of life. Flu is a classical androgen receptor antagonist that can directly bind to androgen receptors in prostate cancer cells, inhibit the production of prostate specific antigen (PSA), induce apoptosis, and suppress tumor growth [6]. However, due to the low targetability of local lesions, systemic administration of androgen receptor antagonists may cause significant fluctuations in hormone levels in the body, and therefore palpable side effects may occur after taking the drugs, such as cardiovascular disease, hypohemoglobinemia, osteoporosis, and psychological disorders [7]. Although ADT can prominently postpone prostate cancer progression for a few years, this treatment approach will eventually become ineffective as patients progress to the advanced castration resistance stage, which is fatal and incurable.

Interestingly, current several researches show major mechanisms related to ADT resistance, and reveal the reasons for entering advanced stage. Several research studies have discovered that tumor-associated macrophages (TAMs) directly contributed to the development of drug resistance in castration treatment for prostate cancer [8]. TAMs are the crucial infiltrating components in the TME, playing critical functions in angiogenesis, remodeling of the extracellular matrix, proliferation of cancer cells, metastasis, suppression of the immune system, as well as resistance to chemotherapy and immunotherapy targeting checkpoint blockade [9]. M1-like TAMs exhibit anti-tumor effects by producing pro-inflammatory cytokines and active substances that kill tumor cells [10]. M2-like TAMs, on the other hand, demonstrate pro-tumor effects through the expression of factors that dampen inflammation, facilitating tumor growth and inhibiting the immune response against tumors [11]. Previous studies have noted an elevated presence of M2-like TAMs within tumor tissues, with their numbers escalating as the tumor advances [12]. Other studies illuminated a correlation between the activation of nuclear factor kappa B (NF-κB) signaling pathway and the development of advanced castration resistance [13]. NF-κB plays a central transcriptional regulatory role on multiple genes, participating in various biological processes such as immunity, cell proliferation, apoptosis, and tumor infiltration [14]. Among the NF-κB families, the p65 protein is a crucial regulator to activate the NF-κB signaling pathway, with the phosphorylated p65 (p-p65) serving as its activated state [15]. When the NF-κB signal pathway is activated, the nuclear translocation of p-p65 will be enhanced, which results in tumor growth and development [16]. Therefore, it is vital to reprogramme M2-like TAMs to M1-like TAMs and inhibit NF-κB pathway in order to reverse the tumor-mediated immunosuppression state and remodel cancer drug resistance.

Cell membrane vesicles (CVs) refer to nanoscale membrane vesicles with a phospholipid bilayer structure originating from cells [17]. Compared to other nanodrug delivery carriers such as liposomes, polymer nanoparticles, metal nanoparticles, and carbon nanomaterials, CVs have unique superiority as a new type of drug delivery system. CVs have low immunogenicity as they originate from the organism itself, which can prevent premature reductive elimination from the body [18]. At the same time, CVs have the ability to cross biological barriers, good biocompatibility, capacity for long-distance transport, high drug loading capacity, and effective accumulation at tumor sites, making them a promising nanocarrier for drug delivery [19]. The probiotic strain Escherichia coli Nissle 1917 (EcN) has been shown to successfully convert tumor-promoting M2-like TAMs into tumor-suppressing M1-like TAMs [20]. Outer membrane vesicles (OMVs) of bacteria are tiny vesicles produced by bacteria that have therapeutic effects similar to the original bacteria [21]. Due to the structural similarities shared with the producer organization, OMVs can retain certain characteristics similar to bacteria themselves [22]. Not only can OMVs derived from EcN be used as the tumor-targeting drug delivery vehicle, but also they can effectively reprogramme M2-like TAMs to M1-like TAMs [23]. EGCG, as a tea polyphenol substance, has been widely used in clinical treatment and is a potential anticancer drug for development [24]. EGCG has been testified to inhibit tumor growth by suppressing the NF-κB pathway [25]. A number of studies in this area have confirmed that EGCG has the capability to hinder the growth of cells in prostate cancer, diminish the levels of androgen receptors within prostate cancer cells, suppress the secretion of PSA by them, trigger the death of such cells, and weaken the resistance of prostate cancer to Flu [26]. Preclinical studies and early-stage clinical trials have demonstrated that EGCG can safely and effectively inhibit the progression of prostate cancer [27]. 

**Fig. 1:**
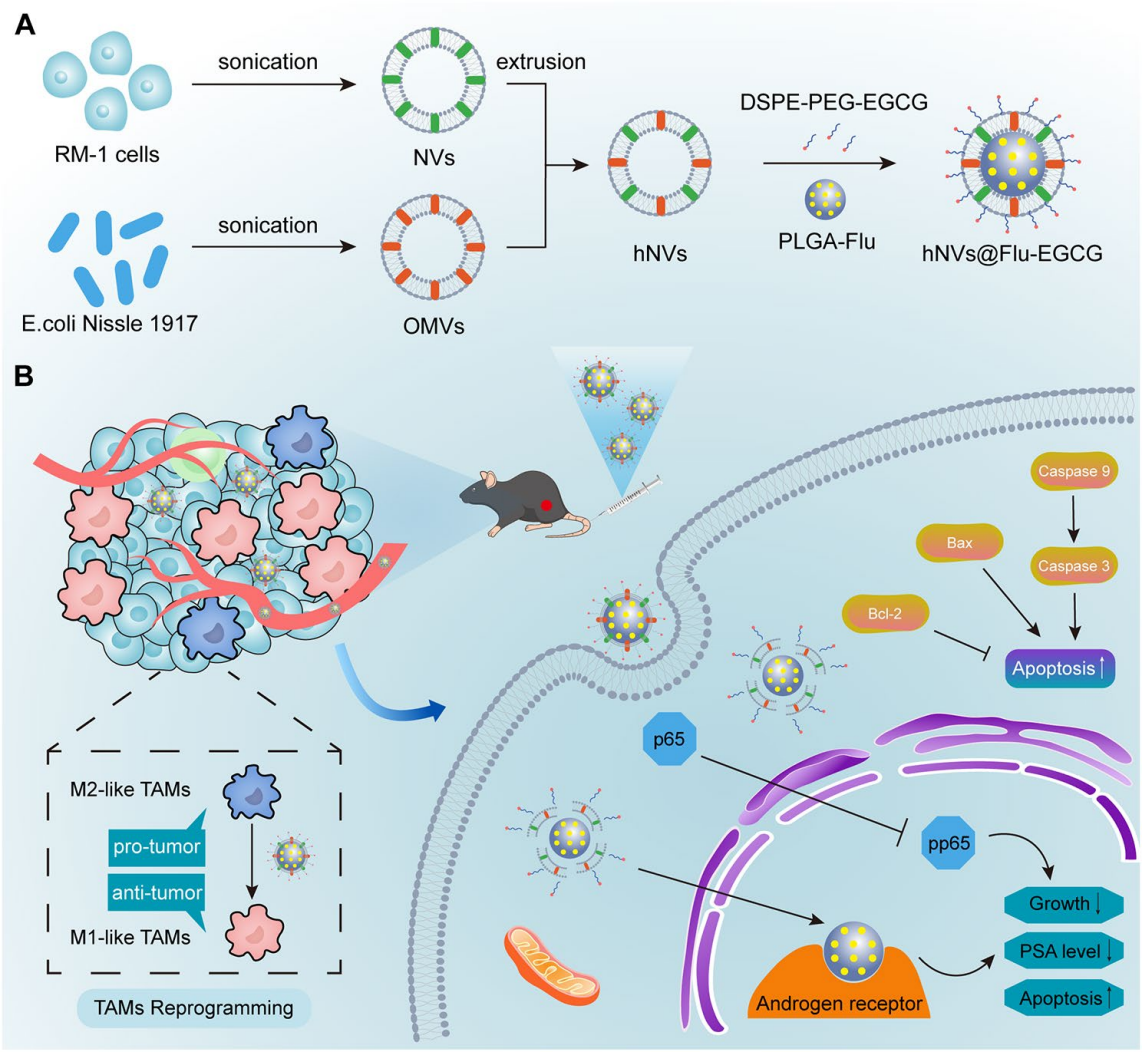
Schematic illustration of preparation and mechanism of hNVs@Flu-EGCG. (**A**) EGCG-modified cell membranes-derived nanoparticles are used as vehicles for targeted delivery of Flu. (**B**) The hNVs@Flu-EGCG target tumor sites, reprogramme M2 macrophages to M1 macrophages, act on androgen receptors to inhibit tumor proliferation, promote apoptosis by inhibiting the NF-κB pathway, reduce PSA level in the body, improve tumor drug resistance and enhance combination therapy

In this study, we report hNVs@Flu-EGCG that can be used for the immunotherapy and ADT combination therapy of prostate cancer. EGCG-modified cancer cells and probiotics-derived nanoparticles are used as vehicles for targeted delivery of Flu to tumor sites (Fig. 1A). Flu acts on androgen receptors to inhibit proliferation of prostate cancer cells. We show that hNVs@Flu-EGCG, which inherit the major capabilities from source cells, efficiently reprogramme TAMs toward M1 phenotype to inhibit tumor growth and suppress NF-κB pathway to promote cancer cells apoptosis (Fig. 1B). More importantly, hNVs@Flu-EGCG clearly reverse tumor immunosuppressive microenvironment and improve tumor drug resistance, thus enhance antitumor properties and reduce non-targeted related side effects. Through the combination therapy, hNVs@Flu-EGCG conspicuously reduce the mouse tumor sizes and prolong overall mouse survival. These unique features of hNVs@Flu-EGCG contribute to improving the drug loading efficiency and strengthen the treatment effects. We foresee that hNVs@Flu-EGCG, which have the excellent biocompatibility, will provide a novel and effective strategy for cancer combination therapy.

## Materials and methods

### Materials

Flutamide, (-)-Epigallocatechin Gallate (EGCG), 3-Aminophenylboronic acid were purchased form Aladdin (Shanghai, China). DSPE-PEG-NHS was purchased from Xi’an Qi Yue Biotechnology Co., Ltd (Xi’an, China). Poly (D, L-lactide-co-glycolic acid) (PLGA 50:50 M = 24,000) was purchased from Macklin (Shanghai, China). The cell membrane mask Alexa647-conjugated wheat germ agglutinin (W32466) was purchased from ThermoFisher Scientific. 4′,6-diamidino-2-phenylindole dihydrochloride (DAPI) were purchased from Sigma-Aldrich. Recombinant Murine IL-4 was purchased from ThermoFisher Scientific.

### Cells and animals

RM-1 cells and RAW 264.7 cells were cultured in Dulbecco’s Modified Eagle’s Medium (DMEM, Invitrogen) supplemented with 10% Fetal Bovine Serum (FBS) and 1% antibiotics (penicillin/streptomycin) at 37 °C in a humidified 95% air/5% CO2 incubator (ThermoFisher Scientific, USA). C57BL/6 mice (male, 6–8 weeks) were bought from Viton Lever Laboratory Animal Technology Co., Ltd (Beijing, China), and the animal study was subject to approval by the Institutional Animal Care and Use Committee of Shenzhen Bay Laboratory.

### Synthesis of PLGA-Flu

PLGA-Flu was prepared using the emulsion-diffusion-solvent evaporation method. 40 mg PLGA and 4 mg Flu were mixed in 2 mL of acetone: dichloromethane (1:4) mixture. Then, 4 mL of PVA aqueous solution (20 mg/mL) was combined with the mixture. The oil-in-water emulsion formed was further sonicated using a sonicator for 20 min. Following this, the homogeneous emulsion was centrifuged at 20,000 g for 40 min. The supernatant was then subjected to HPLC analysis to determine the free drug content, while the sediment underwent two washes with PBS. For the HPLC analysis, we used a mobile phase with water/methanol. Water phase: organic phase (40%/60% v/v) at a flow rate of 1.0 mL/min. The injection volume was 20 µL, and the detection wavelength was 230 nm. The Flu drug-loading capacity (DLC) and drug encapsulation efficiency (DEE) were evaluated by formula below: DLC% = (m total - m free) / m total × 100%; DEE% = (m total – m free) / m sediment × 100%. Here, m total represents the total amount of Flu, m free represents the free amount of Flu in supernatant, and m sediment represents the amount of sediment after free-drying.

### Synthesis of DSPE-PEG-EGCG

The method used for synthesizing DSPE-PEG-EGCG was adapted from a previously documented procedure with slight adjustments. In brief, DSPE-PEG2000-NHS and 3-Aminophenylboronic acid (DSPE-PEG2000-NHS:3-aminophenylboronic acid: 2/1, mol/mol) were dissolved in 10 mL of N, N-Dimethylformamide (DMF). The reaction mixture was stirred gently at room temperature for 36 h. Then, the mixture was dialyzed extensively (dialysis bag Mw cutoff 2,000 Da) in distilled water for 48 h to eliminate any impurities and then lyophilized 48 h to produce DSPE-PEG-phenylboronic acid. After that, DSPE-PEG-phenylboronic acid and EGCG (DSPE-PEG-phenylboronic acid: EGCG: 2/1, mol/mol) were dissolved in 10 mL of anhydrous ethanol. The reaction mixture was stirred gently at room temperature for 36 h. Then, the mixture was dialyzed extensively (dialysis bag Mw cutoff 2,000 Da) in distilled water for 48 h to eliminate the remaining impurities and then lyophilized 48 h to obtain DSPE-PEG-EGCG. The characterization of the synthesized product is conducted through 1 H NMR analysis using a Bruker 400 MHz NMR spectrometer in DMSO-d6 solvent.

### Preparation and characterization of hNVs@Flu-EGCG

**(i)** RM-1 cells were collected and then suspended in the PBS buffer. The cells were then disrupted 5 min at 40% power using the ultrasonic disruptor. After fragmentation, the supernatant was gathered through centrifugation at 4,000 g for a duration of 40 min, while the membranes were isolated via centrifugation at 100,000 g for a period of 1 h on the Beckman Coulter Optima XE-90 ultracentrifuge to obtain cell membrane particles. The cellular membrane pellets were gathered and resuspended in PBS buffer. Following this, they were extruded through polycarbonate porous membranes of varying sizes (800 nm, 400 nm, and 200 nm) utilizing a mini extruder provided by Avanti Polar Lipids, allowing for the isolation of NVs. **(ii)** In order to isolate EcN OMVs, bacteria were introduced to 400 mL of LB broth and incubated at 37 °C with shaking (200 rpm) for a period of 48 h. Subsequently, the bacterial cells were removed from the culture via centrifugation at 5,000 g for 20 min at 4 °C, and the resulting supernatant was filtered using a 0.45 μm filter. The resulting concentrated solution underwent further centrifugation at 150,000 g for 2 h at 4 °C using a Beckman Coulter Optima XE-90 ultracentrifuge to harvest the OMVs. The OMVs were then retrieved and suspended in PBS buffer. Following this, the NVs and OMVs were combined at a protein weight ratio of 1:2 and extruded through 200-nm pores on a mini extruder in order to produce hNVs. PLGA-Flu and hNVs were mixed (PLGA-Flu mass weight: hNVs protein weight:1:1) and treated with a water-bath ultrasound for 30 min to obtain hNVs@Flu. After that, DSPE-PEG-EGCG and hNVs@Flu (DSPE-PEG-EGCG mass weight: hNVs@Flu protein weight:1:5) were co-inoculated at 37 °C for 30 min to obtain hNVs@Flu-EGCG. **(iii)** The nanoparticles’ sizes and zeta potentials were measured utilizing Dynamic light scattering (DLS; Zetasizer Nano ZS, Malvern Instruments) and NanoSight NS300 (Malvern Instruments). Additionally, the nanoparticles’ morphology was examined with transmission electron microscopy (JEM-2100).

### SDS-PAGE

For sodium dodecyl sulfate-polyacrylamide gel electrophoresis (SDS-PAGE), OMVs, NVs hNVs and hNVs@Flu-EGCG were added into the protein extraction buffer, and followed by protein concentration measurement using a bicinchoninic acid (BCA) protein quantification kit (KeyGEN BioTECH, China), and the protein was denatured by heating the samples at 100 °C for 5 min Subsequently, equal amounts of proteins were loaded into a 10% SDS-polyacrylamide gel and separated based on the specific parameters (80 V for 30 min and 120 V for 90 min). After electrophoresis, the gel was stained with Commassie blue (Solarbio, China) for 30 min, washed with DI water and decolorized three times before the observation.

### Cell toxicity assays

The CCK8 assay was conducted to do the cytotoxicity test. Initially, Plating RM-1 cells onto 96-well polystyrene plates at a density of 10,000 cells per 100 µL of medium per well. Following a 12 h incubation at 37 °C, the cells were exposed to varying concentrations of blank PLGA, Flu, PLGA-Flu, OMVs, hNVs, hNVs@Flu, hNVs-EGCG, and hNVs@Flu-EGCG in the medium for 24 h. Subsequently, the cells were incubated with 100 µL of CCK8 solution at 37 °C for 2 h. Cell viability was assessed by measuring the absorbance at 450 nm using a microplate reader.

### Live/dead cell staining assay

To conduct the live/dead cell staining experiment, RM-1 cells were plated in 6-well plates at a density of 10,000 cells per well. They were then treated with PBS, PLGA-Flu, hNVs, hNVs-EGCG, and hNVs@Flu-EGCG (Flu at a concentration of 20 µg/mL) for 24 h. Following treatment, the cells were incubated with the Calcein AM/PI assay working solution in the absence of light for 30 min, and subsequently imaged using CLSM.

### Cell colony formation assay

RM-1 cells were seeded in 6-well plates at 5,000 cells per well and cultured for 96 h, and then incubated with PBS, PLGA-Flu, hNVs, hNVs-EGCG and hNVs@Flu-EGCG (Flu, 20 µg/mL) for other 72 h. After replacing fresh medium, cells were continued to be cultured until a sufficiently large colony was formed, and then the colony formation was analyzed by calculating after fixing with paraformaldehyde and staining with crystal violet reagent (Beyotime, Shanghai, China).

### Hemolysis test

Blood was collected from the healthy male C57BL/6 mice and placed in anticoagulant tubes containing EDTA for 1 h. Then, the blood underwent centrifugation at 1,200 rpm for 5 min and was then suspended in PBS buffer a total of three times until the color of the supernatant was no longer visible. Following this, the blood cells were exposed to varying levels of hNVs@Flu-EGCG for a duration of 5 h, with distilled water serving as a positive control and PBS as the negative control. Subsequently, the supernatant was retrieved through centrifugation at 15,000 rpm for 15 min, and its absorbance at 570 nm was measured using a microplate reader. The hemolysis ratio (HR) was calculated by the following formula: HR = (sample absorbance – negative absorbance) / (positive absorbance − negative absorbance) × 100%.

### Apoptosis assay

The Annexin V-FITC/PI Apoptosis Detection Kit (Dojindo, Japan) was utilized to perform the apoptosis assay and data analysis was done by FACS flow cytometry. Cells were initially plated in 6-well plates with 5 × 10^5^ cells per well and cultured for 12 h. Subsequently, different treatments including PBS, PLGA-Flu, hNVs, hNVs-EGCG, and hNVs@Flu-EGCG (with Flu at 20 µg/mL) were administered to the cells for a period of 24 h. After the incubation, the cells were collected and stained with Annexin-V FITC and PI as per the provided guidelines. Flow cytometry was employed to analyze the stained cells, and the resultant data was processed using FlowJo software.

### *In vitro* cellular uptake of hNVs@Flu-EGCG 

Flow cytometry (CytoFLEX LX, Beckman Coulter) and LSM900 (Zeiss) were used to measure the cellular uptake of hNVs@Flu-EGCG. hNVs@Flu-EGCG were labeled with fluorescent dyes 3,3′-dioctadecyloxacarbocyanine perchlorate (DiO). For flow cytometry analysis, RM-1 cells were seeded in 6-well plates and incubated with DiO-labeled hNVs@Flu-EGCG for 0, 1, 2, 4, 8, 12 and 24 h. After that, the cells underwent a series of preparatory steps which included being collected, washed thrice with PBS, and suspended in 300 µL of FACS buffer for flow cytometry analysis. In the context of fluorescence imaging analysis, RM-1 cells were first seeded in the confocal dish and allowed to adhere for a period of 12 h. Subsequently, DiO-labelled hNVs@Flu-EGCG in fresh DMEM were added to the cells and incubated for the same duration as described earlier. The cells were then fixed using 4% paraformaldehyde for 15 min and subjected to another round of triple PBS washes. Following this, the nucleus was stained with DAPI and the cell membrane was stained with Alexa647-conjugated wheat germ agglutinin (WGA) before observation using LSM900.

### Western blot

After 24 h of culturing RM-1 cells in 10 cm dishes, the medium was replaced with the fresh ones containing PBS, PLGA-Flu, hNVs, hNVs-EGCG, and hNVs@Flu-EGCG (Flu, 20 µg/mL), and they were cultured for an additional 24 h. Subsequently, the collected cells were subjected to total protein analysis. The denatured samples were loaded onto a 10% SDS-PAGE gel and transferred to polyvinylidene fluoride (PVDF) membranes, which were then blocked with 5% nonfat milk at room temperature for an hour, followed by incubation with primary antibodies: GAPDH (5174, 1:1000), Caspase-9 (9508, 1:1000), Caspase-3 (14,220, 1:1000), Cleaved caspase-3 (9664, 1:1000), Bcl-2 (ab182858, 1:1000), Bax (ab32503, 1:1000), PSMA (ab314142, 1:1000), androgen receptor (A19611, 1:1000), p-65 (ab32536, 1:1000), pho-p65 (ab76302, 1:1000) at 4 °C overnight. Following the completion of the initial steps, the PVDF membranes were washed thrice with Tris-buffered saline with Tween 20 (1× TBST). Subsequently, they were incubated with a secondary IgG-horseradish peroxidase (HRP) antibody for a duration of 2 h at room temperature. This was followed by another round of washing with 1× TBST. The blots were developed by using a chemiluminescence detection system (Tanon 5200 Multi, Shanghai, China).

### *In vivo* biotoxicity evaluation 

Healthy C57BL/6 mice (6–8 weeks old) were divided into five groups at random and received intravenous injections of PBS (as the control), PLGA-Flu, hNVs, hNVs-EGCG, and hNVs@Flu-EGCG (Flu, 10 mg/kg). On day 7 post-injection, all mice were euthanized. The major organs, including heart, liver, spleen, lung, and kidney were collected, fixed in 4% paraformaldehyde, sectioned, and then stained with hematoxylin-eosin (H&E) for histopathological examination (Servicebio, Wuhan, China). Additionally, blood samples were obtained from the mice for the purpose of performing both blood biochemistry and blood routine analysis including alanine transaminase (ALT), aspartate aminotransferase (AST), alkaline phosphatase (ALP), blood urea nitrogen (BUN), white blood cell (WBC), red blood cell (RBC), hemoglobin (HGB), hematocrit (HCT), mean corpuscular volume (MCV), mean corpuscular hemoglobin (MCH), mean corpuscular hemoglobin concentration (MCHC), and platelets (PLT).

### *In vivo* tumor targeting 

PLGA-Flu, OMVs@Flu and hNVs@Flu were labelled with fluorescent dyes 1,1’-dioctadecyl-3,3,3’,3’-tetramethylindotricarbocyanine iodide (DiR) to investigate the in vivo fluorescence imaging. The RM-1 cells (5 × 10^5^ cells) were injected subcutaneously into the right thighs of C57BL/6 mice (6–8 weeks old) to establish a mouse model of subcutaneous prostate cancer. Once the tumor volume reached around 100 mm^3^, DiR-labelled PLGA-Flu, OMVs@Flu, and hNVs@Flu were administered to RM-1 tumor-bearing mice via intravenous injection. Following this, imaging was conducted at specific time points (0, 2, 4, 8, 12, 24, and 48 h) using the IVIS imaging system (PerkinElmer, USA) to evaluate the distribution within the body. Post imaging, the mice were humanely euthanized, and key organs, including the heart, liver, spleen, lungs, kidney, and tumor were harvested for subsequent in vitro imaging analysis after 48 h. Furthermore, the organs and tumor tissues were processed, sliced, and stained to facilitate additional fluorescence imaging analysis in vitro.

### *In vivo* antitumor capabilities

The RM-1 cells (5 × 10^5^ cells) were injected subcutaneously into the right thighs of C57BL/6 mice (6–8 weeks old). Once the tumor volume of RM-1 tumor-bearing mice grew to 100 mm^3^-200 mm^3^, the mice were categorized into five groups at random and were intravenously injected with PBS (as control), PLGA-Flu, hNVs, hNVs-EGCG and hNVs@Flu-EGCG (Flu, 10 mg/kg) once every other day for five times. Monitoring of tumor size and body weight of the mice occurred every other day. The part of the mice was sacrificed, and the tumors were extracted, fixed using 4% paraformaldehyde, sectioned, and stained with Ki-67 and TUNEL to investigate the impact of the different treatments on tumor cell proliferation and apoptosis (Servicebio, Wuhan, China). In order to assess the antitumor capabilities of nanoparticles, tumors were taken from each group to be photographed on the last day after treatment (*n* = 5 per group). At the same time, mice blood samples were collected to detect PSA level using a Prostate Specific Antigen ELISA kit (nenbioscience, China). For the survival study (*n* = 5 per group), mice were considered dead once the tumor volume exceeded 1500 mm^3^. Tumor inhibition rate (TIR) was worked out by the formula beneath. TIR (%) = (Vc − Vt) /Vc × 100%. Here, Vc represents the mean tumor volume of control group; Vi represents the mean tumor volume of different treatment groups.

### *In vivo* tumor immune microenvironment analysis

The tumors were processed following the sacrifice of the RM-1 tumor-bearing mice. The tumors were excised, cut into small pieces, and then subjected to an incubation process with RPMI 1640 media containing collagenase IV (0.2 mg/mL), DNase I (250 U mg/mL) and hyaluronidase (0.1 mg/mL) for a duration of 40 min at 37 ℃. Subsequently, the homogenates underwent filtration through a 70 μm nylon mesh to obtain single-cell suspensions which were then washed with PBS. The cells were further treated with fluorescence-labelled antibodies: CD45 (APC-Cy7; BD, USA), CD11b (FITC; BD, USA), F4/80 (PE-Cy7; BD, USA), CD80 (Pacific Blue; BD, USA), CD206 (Alexa Fluor 647; BD, USA). All antibodies were diluted appropriately and incubated with the cells under specific temperature conditions as per the provided instructions. Subsequently, the samples obtained were assessed through flow cytometry methods. Figure S11 (Supporting Information) provides comprehensive details on flow cytometric gating and sorting approaches. For ELISA assay, RM-1 tumor tissues were harvested to detect cytokine levels. The intratumor levels of TNF-α, IL-6, and IL-10 were detected with ELISA kits (Servicebio, Wuhan, China). For immunofluorescence staining, Tumor tissues were harvested and fabricated into the paraffin section and then were stained with the anti-CD206 antibody (red), anti-CD80 antibody (green), and DAPI (blue) (Servicebio, Wuhan, China).

### Statistical analysis

All data are presented as mean ± standard deviation (S.D.) unless otherwise noted. Statistical significance was analyzed using one-way (or 2way) ANOVA with a Tukey’s test (or with a Dunnett’s test). GraphPad Prism software was used to analyze the data. Statistical significance was defined as *P* < 0.05.

## Results

### Preparation and characterization of hNVs@Flu-EGCG

**Fig. 2:**
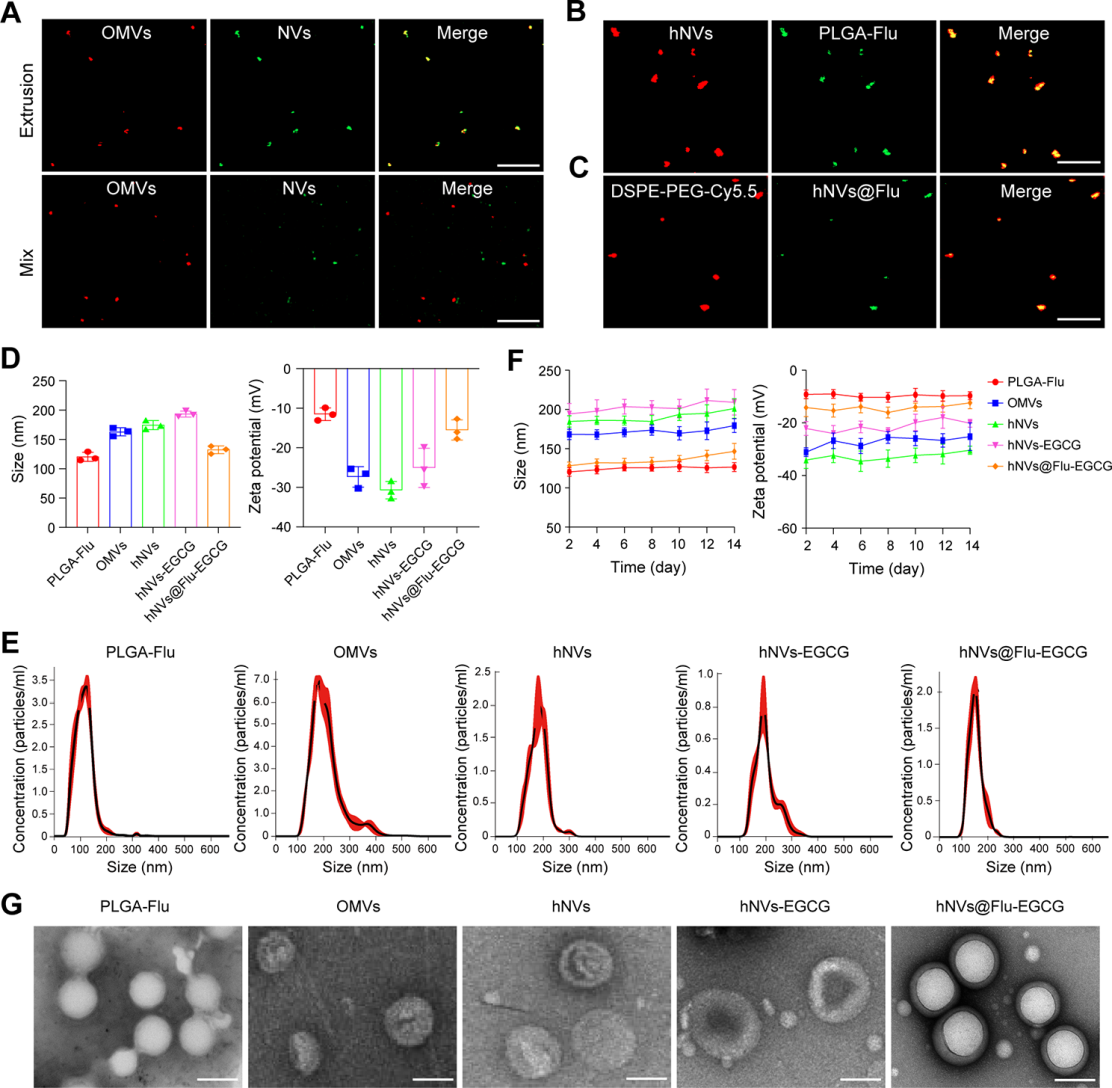
Preparation and characterization of hNVs@Flu-EGCG. (**A**) Confocal fluorescence images of hNVs. Red, OMVs; green, NVs. Scale bar, 5 μm. (**B**) Confocal fluorescence images of hNVs@Flu. Red, hNVs; green, PLGA-Flu. Scale bar, 5 μm. (**C**) Confocal fluorescence images of hNVs@Flu-Cy5.5. Red, DSPE-PEG-Cy5.5; green, hNVs@Flu. Scale bar, 5 μm. (**D**) Size distribution and zeta potential of PLGA-Flu, OMVs, hNVs, hNVs-EGCG and hNVs@Flu-EGCG (*n* = 3). (**E**) NTA results of nanoparticles. (**F**) Stability of nanoparticles over the period in terms of size change at 4 ℃ (*n* = 3). (G) TEM images of nanoparticles. Scale bar, 100 nm

Briefly, the preparation of hNVs@Flu-EGCG includes three steps: (1) obtaining nanovesicles (NVs) derived from RM-1 cells and OMVs derived from EcN, (2) fusing NVs and OMVs to form the hNVs, and (3) obtaining EGCG-modified and Flu-loaded nanoparticles called hNVs@Flu-EGCG. Firstly, NVs were generated through extrusion and OMVs were generated through ultracentrifugation. Then, the resulting NVs and OMVs were mixed and co-extruded through nanopores repeatedly to form hNVs. To further determine if NVs and OMVs were fused successfully, OMVs were labeled with fluorescent dyes 1,1’-Dioctadecyl-3,3,3’,3’-tetramethylindocarbocyanine perchlorate (DiI) and NVs were labelled with fluorescent dyes 3,3′-dioctadecyloxacarbocyanine perchlorate (DiO) before fusion. The observation of hNVs under a confocal microscope revealed a noteworthy occurrence: a noticeable overlap of fluorescent signals, indicating the successful fusion of OMVs and NVs. At the same time, two types of vesicles labelled with different fluorescent dyes were only mixed, followed by observation under the confocal microscope, and no fluorescence overlap was detected (Fig. 2A). In addition, sodium dodecyl sulfate-polyacrylamide gel electrophoresis (SDS-PAGE) was used to further characterize the uploading membrane proteins of different constituents (Figure S1, Supporting Information). Similar protein bands of OMVs and NVs could be found in hNVs and hNVs@Flu-EGCG, which indicated that there was no significant degradation of membrane protein components in the production of nanoparticles.

Earlier researches have shown that EcN could reprogramme M2-like TAMs into M1-like TAMs [28]. To verify if the vesicles derived from EcN exhibited the similar functions, flow cytometry was conducted to analyze RAW 264.7 cells treated with different formulations. RAW264.7 is a mouse macrophage-like cell line, which has been used as an appropriate macrophage model. LPS can drive RAW 264.7 cell polarization toward the M1 macrophage phenotype, and IL-4 can modulate RAW 264.7 cell polarization toward M2 macrophage phenotype. As is shown in Figure S2 (Supporting Information), compared to the control group, after adding OMVs and hNVs@Flu-EGCG, the number of CD80^+^ (M1 macrophages marker) cells increased and the number of CD206^+^ (M2 macrophages marker) cells decreased, which suggested that OMVs and hNVs@Flu-EGCG promoted the transition of M2-like TAMs to M1-like TAMs.

Flutamide is a nonsteroidal antiandrogen drug that can reduce androgen levels in the body used to slow prostate cancer progression. Poly (lactic-co‐glycolic acid) (PLGA), as a nonimmunogenic, noninflammatory, and nontoxic polymer, has been used to prepare biocompatible and biodegradable nanoparticles, thereby improving the solubility and biological activity of drugs. Accordingly, flutamide-loaded PLGA (PLGA-Flu) nanoparticles were prepared by the emulsion-diffusion-solvent evaporation method. The drug-loading capacity (DLC) and drug encapsulation efficiency (DEE) of Flu determined by high-performance liquid chromatography (HPLC) were 81.81% ± 0.19% and 4.71% ± 0.005%, respectively (Figure S3, Supporting Information).

Cell membrane-derived nanovesicles can load drugs and improve encapsulated drugs’ stability and tumor targetability. Usually, nanovesicles were only used to load one drug. Loading two or more drugs has the disadvantages of higher production costs, lower drug-loading capacity, slow drug release rate, and drug interactions [29]. Therefore, EGCG-modified and Flu-loaded nanoparticles were developed. To acquire EGCG-modified nanoparticles, We first prepared DSPE-PEG-EGCG according to previous reports [30]. 3-aminophenylboronic acid react with DSPE-PEG2000-NHS to form DSPE-PEG-phenylboronic acid, EGCG then react with DSPE-PEG-phenylboronic acid to form DSPE-PEG-EGCG through the formation of a boronate ester bond. Their chemical structures were characterized by the Nuclear Magnetic Resonance (1 H NMR) spectrum (Figure S4, Supporting Information). After that, hNVs@Flu-EGCG were prepared via sonication and co-incubation.

To validate if PLGA-Flu were coated by hNVs successfully, PLGA-Flu were labelled with DiO and hNVs were labelled with DiI first. After sonication for 30 min, the fluorescence image of hNVs@Flu showed the green fluorescence of the PLGA-Flu core and the red fluorescence of the membrane around, suggesting PLGA-Flu were coated by hNVs indeed (Fig. 2B). After that, to validate if hNVs@Flu were modified by DSPE-PEG-EGCG, DSPE-PEG-Cy5.5 was synthesized using the same method above. Cyanine 5.5 (Cy5.5) is an anthocyanin fluorescent dye that emits near-infrared (NIR) fluorescence that can be used to label various substances. hNVs@Flu were labeled with DiO and were co-inoculated with DSPE-PEG-Cy5.5 at 37 °C for 30 min. Then the composition was observed using a confocal microscope, with the fluorescence image showing hNVs@Flu showed the green fluorescence and the surrounding DSPE-PEG-Cy5.5 emitting red fluorescence, suggesting hNVs@Flu-EGCG had been successfully prepared (Fig. 2C).

Cell membrane fragments could be made into cell membrane nanovesicles via the extrusion method, and the nanovesicles have good homogeneity, which has already been confirmed in previous studies [31]. The size distribution and zeta potential of PLGA-Flu, OMVs, hNVs, hNVs-EGCG and hNVs@Flu-EGCG were measured by dynamic light scattering (DLS) analysis and nanoparticle tracking analysis (Fig. 2D and E). It could be seen that the sizes of the nanoparticles were approximately 100–200 nm, and they were all negatively charged. the average potential of PLGA-Flu (-11.52 ± 1.60 mV) was reduced compared to that of hNVs@Flu-EGCG (-15.51 ± 2.56 mV), probably due to the successful coating of hNVs onto the PLGA polymer. Afterwards, these nanoparticles underwent continuous evaluation through monitoring of both particle size and zeta potential. The findings indicated that, within buffers, these nanoparticles exhibited stable characteristics for a minimum of two weeks. This observation strongly implied that the structural stability of these nanoparticles was commendable (Fig. 2F). Meanwhile, the morphologies of nanoparticles were analyzed using transmission electron microscopy (TEM). We observed that PLGA-Flu exhibited the white spherical morphological feature, and OMVs, hNVs and hNVs-EGCG displayed a spherical shape. Furthermore, hNVs@Flu-EGCG displayed a clear core–shell nanostructure with PLGA-Flu “core” and hNVs “shell”, suggesting that PLGA-Flu were successfully coated by the nanovesicles (Fig. 2G).

### Antitumor efficacy and toxicity of hNVs@Flu-EGCG *in vitro*

**Fig. 3:**
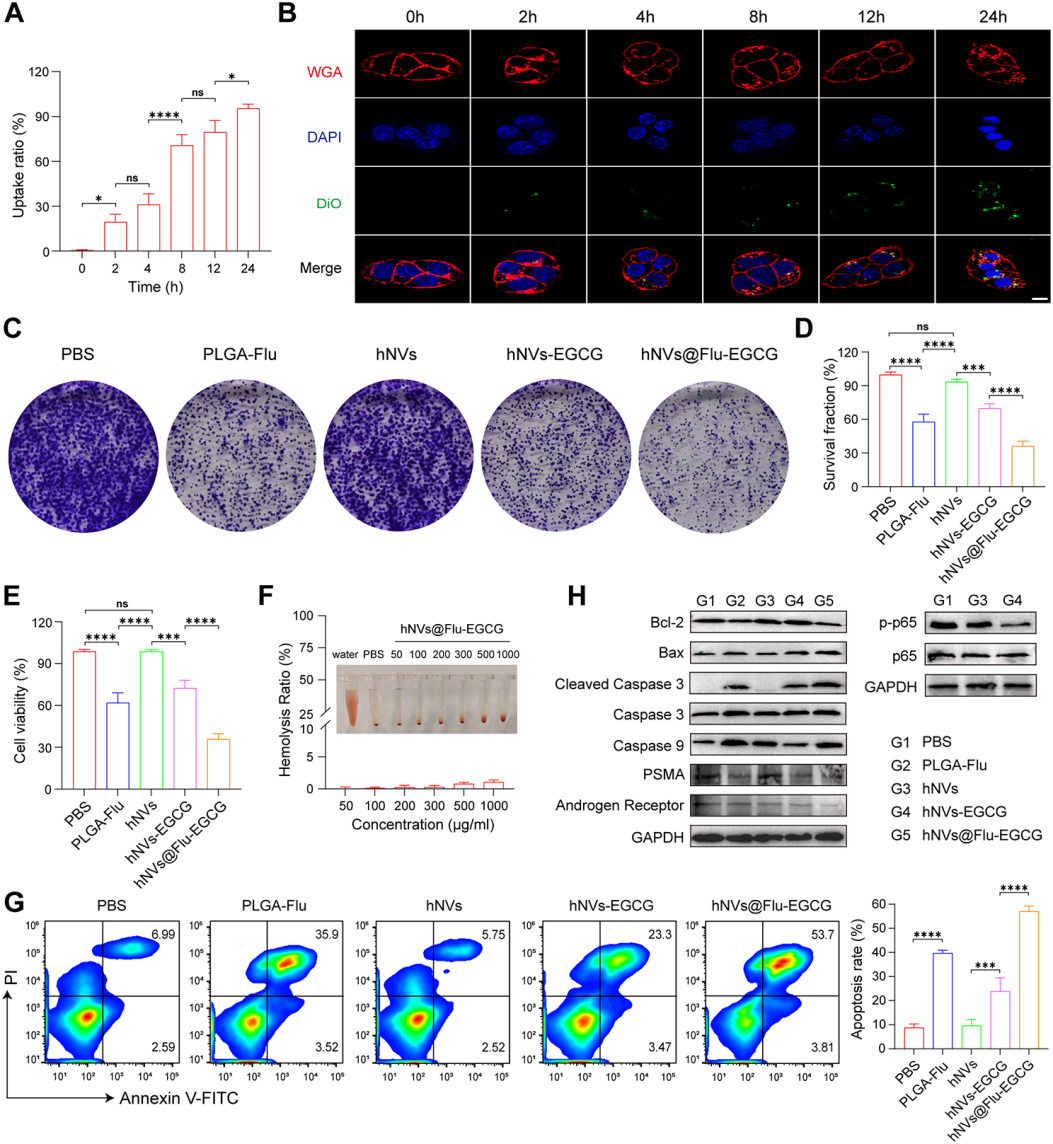
The biological effect induced by nanoparticles in vitro. (**A**) The uptake ratio of hNVs@Flu-EGCG (Flu, 20 µg/mL) in RM-1 cells was analyzed using flow cytometry (*n* = 3). (**B**) Confocal fluorescence images showing cellular uptake of hNVs@Flu-EGCG in RM-1 cells in at different time points. Scale bar, 10 μm. (**C**) Photographs of RM-1 cell colonies from after various treatments. (**D**) The survival fraction of RM-1 cells in cell colony formation assay (*n* = 3). (**E**) Quantification of the ratios of live cells (calcein-AM^+^ PI^−^) in live/dead cell staining assay (*n* = 3). (**F**) Images of hemolysis and the rate of hemolysis following exposure to varying concentrations of hNVs@Flu-EGCG (*n* = 3). (**G**) Apoptosis in cells was assessed by flow cytometry with Annexin-V FITC/PI staining after different types of treatment (*n* = 3). (**H**) Western blot assays demonstrated the activation of the apoptosis pathway and suppression of the NF-κB pathway in RM-1 cells after various treatments. All data are expressed as mean ± S.D. NS: no significance, *P < 0.05, **P < 0.01, ***P < 0.001, ****P < 0.0001

To evaluate the cell uptake efficiency of hNVs@Flu-EGCG in RM-1 cells, Flow cytometry and confocal laser microscopy (LSM900) were used in vitro. First of all, flow cytometry was performed to assess the uptake of hNVs@Flu-EGCG. As we can see in Fig. 3A and Figure S5 (Supporting Information), it is evident that following incubation of RM-1 cells with nanoparticles for a specific period of time, the ratio of cellular uptake increased as the incubation time extended. The peak uptake ratio was observed after 24 h. Subsequently, to visualize cellular uptake and intracellular distribution, hNVs@Flu-EGCG were labelled with DiO and DAPI-stained nuclei in blue. Observation made using a confocal microscope indicated that the internalization of nanoparticles in RM-1 cells increased with co-culture time, which is consistent with the flow cytometry results (Fig. 3B).

The cytotoxicity assessment of nanoparticles in vitro was carried out using the CCK8 assay, revealing an increase in cytotoxicity with the augmentation in hNVs@Flu-EGCG concentration (Figure S6, Supporting Information). Subsequently, a cell colony formation assay was conducted to evaluate the proliferation ability of RM-1cells, and the result manifested that the number of cell colony formations decreased after treatments of nanoparticles compared to the control group (Fig. 3C and D). At the same time, RM-1 cells were treated with Calcein acetoxymethyl ester (Calcein-AM) and Propidium Iodide (PI) to differentiate between living and dead cells following the application of different formulations. Calcein-AM is a fluorescent dye that can pass through cell membranes and can be hydrolyzed into Calcein inside living cells, resulting in green fluorescence. PI is a nucleic acid dye that can only penetrate the membranes of dead cells and bind to DNA, producing red fluorescence. As is shown in Fig. 3E and Figure S7 (Supporting Information), we found that RM-1 cells treated with PLGA-Flu, hNVs, hNVs-EGCG, and hNVs@Flu-EGCG were inhibited in growth, and hNVs@Flu-EGCG group exhibited the most substantial inhibitory effect on cell viability.

Biomimetic membrane-coated nanomaterials are universally known because of their remarkable biocompatibility and prolonged blood circulation time [32]. A hemolysis assay was performed to evaluate the hemocompatibility of hNVs@Flu-EGCG, and the result demonstrated no significant hemolysis rate for these nanoparticles even at higher concentrations as the hemolysis rate was less than 5%, which indicated satisfactory hemocompatibility (Fig. 3F). Furthermore, after various interventions, cells were stained with Annexin V-FITC and propidium iodide (PI) using the Annexin V-FITC/PI Apoptosis Detection Kit. Flow cytometry was then utilized to analyze cell apoptosis. The findings revealed that nanoparticles exhibited a stronger capacity to induce RM-1 cells apoptosis and showed the efficacious tumor inhibition effect in vitro (Fig. 3G).

We further investigated the molecular mechanism by which nanoparticles inhibited proliferation and promoted apoptosis of tumor cells via western blot analysis (Fig. 3H). Both Caspase 3 and Caspase 9 belong to the cysteine protease family and play cardinal roles in the process of cell apoptosis. During the process of cell apoptosis, Caspase 3 is activated and participates in the degradation of intracellular proteins. Cleaved caspase 3 is a lytic product of activated Caspase 3, which can induce cell apoptosis. Caspase 9 triggers cell apoptosis by activating other caspase proteases. The membrane protease known as prostate specific membrane antigen (PSMA) is usually found in abundance on the surface of prostate cancer cells, and androgen receptor expression is intimately related to proliferation of cancer cells. When the growth of prostate tumors is inhibited, the expression level of PSMA and androgen receptor decreases. In this study, we found that the treatment groups exhibited increased apoptosis and decreased proliferation of RM-1 cells compared to the control group, in which the hNVs@Flu-EGCG group elicited the most potent effect. Simultaneously, compared with untreated RM-1 cells, the expression of Bax protein that can promote apoptosis increased in treatment groups, and the expression of anti-apoptotic Bcl-2 protein correspondingly decreased. Furthermore, it is reported that EGCG can restrain the activation of NF-κB signal pathway, inhibit the generation of p65, thereby promoting the apoptosis of tumor cells and reducing drug resistance. Since the p-p65 is the active form of this protein, western blot analysis was performed to analyze the NF-κB protein level. We found that the total protein level of p65 did not change distinctly, elucidating that EGCG may function through inhibiting its phosphorylation instead of affecting the total protein, and the hNVs-EGCG group measurably downregulated the expression of p-p65 protein compared to hNVs group and control group.

### Biosafety and tumor targeting capability of nanoparticles *in in vivo*

Next, we evaluated the biosafety of nanoparticles *in vivo.* Hematoxylin-eosin (H&E) staining for organ sections of different groups suggested no substantial damage to the heart, liver, spleen, kidneys, and lungs (Figure S8, Supporting Information). After receiving different treatments, the mice were sacrificed. Blood samples were then collected for routine blood tests and biochemistry examinations. These nanoparticles all exhibited a negligible impact on the ALT, AST, ALP and BUN levels, compared to those of the PBS group (Figure S9, Supporting Information). Overall, these findings consistently demonstrate the good biosafety, biocompatibility, and low side effects of the nanoparticles.

**Fig. 4:**
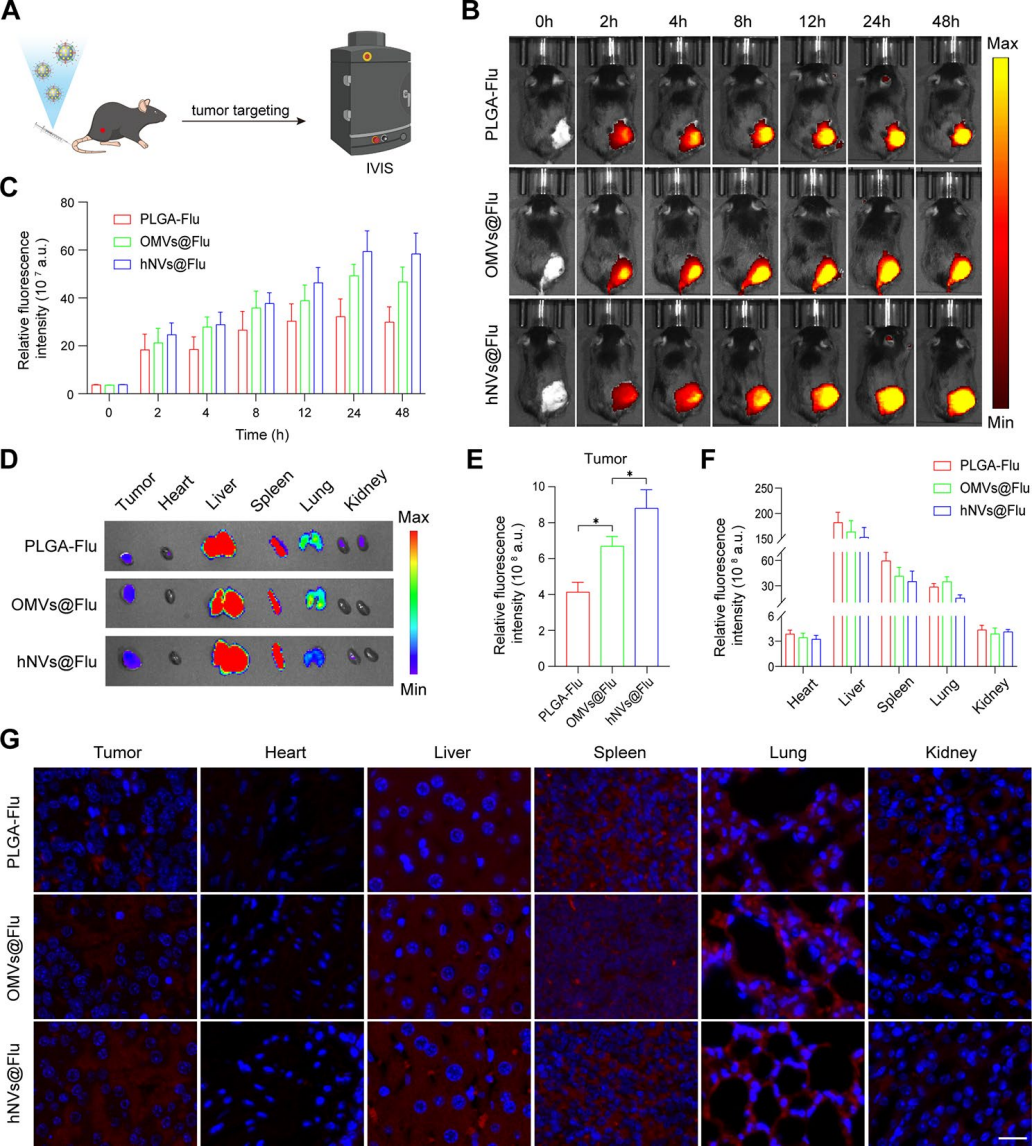
Tumor targeting capability of nanoparticles in vivo. (**A**) Diagrammatic sketch of tumor targeted nanoparticles labelled with fluorescence through intravenous injection. (**B**) Biodistribution of RM-1-bearing mice in PLGA-Flu, OMVs@Flu, and hNVs@Flu groups at various time intervals by IVIS. (**C**) The relative fluorescence intensity of PLGA-Flu, OMVs@Flu, and hNVs@Flu groups was quantified at various time intervals (*n* = 3). (**D**) The major organs and tumors were gathered after 48 h and investigated through IVIS imaging. (**E**) Quantification of the relative fluorescence intensity of the tumors and major organs (*n* = 3). (**F**) Representative fluorescence images of the sections of tumors and major organs. Scale bar, 20 μm. All data are expressed as mean ± S.D. NS: no significance, * *P* < 0.05, ** *P* < 0.01, *** *P* < 0.001, **** *P* < 0.0001

It is well acknowledged that cell membrane-camouflaged biomimetic nanoparticles endow them with terrific immune escape and homologous targeting capabilities [33]. The peculiar targeting ability of nanoparticles toward tumor tissue is exceedingly impressive. Previous work indicated that natural biological membrane-derived nanovesicles had low immunogenicity, high tumor targeting ability and high controllability of smart nanocarrier design, which can enhance the accumulation of inner drugs in tumor sites [34]. For the purpose of detecting the tumor-targeting capability of nanoparticles, the DiR-labelled PLGA-Flu, OMVs@Flu and hNVs@Flu were administered intravenously into the mice with RM-1 tumors, and their fluorescence was captured using an IVIS spectrum imaging system at different time points respectively (Fig. 4A). As shown in Fig. 4B and C, the fluorescence intensity at the tumor sites increased and reached a peak at 24 h along with the injection time increased, and hNVs@Flu showed higher effective accumulation in tumor sites than PLGA-Flu and OMVs@Flu.

After 48 h intravenous injection, mice were euthanized to analyze the fluorescence intensity in diverse organs (Fig. 4D, E and F). The results suggested that the accumulation of OMVs@Flu and hNVs@Flu was much higher than that of PLGA-Flu, which indicated that natural biological membrane camouflage improved the accumulation of nanoparticles in the tumor sites than inorganic materials. The quantitative analysis was conducted to compare the relative fluorescence intensity in tumor sites between different types of nanoparticles. Specifically, there was a 2.13 and 1.62-fold increase in relative fluorescence intensity for hNVs@Flu and OMVs@Flu, respectively, in comparison to PLGA-Flu. Furthermore, there was a 1.32-fold increase in relative fluorescence intensity for hNVs@Flu compared with OMVs@Flu, which indicated that homologous cancer cell membrane-derived nanoparticles exhibited superior homologous tumor targeting capabilities. Subsequently, the tumor-targeting capabilities of these DiR-labelled nanoparticles were established using a fluorescence microscope (Fig. 4G). The greater presence of the red fluorescence signal indicating the dispersion of DiR-labelled nanoparticles was observed in the hNVs@Flu group compared to both the PLGA-Flu and OMVs@Flu groups, aligning with the aforementioned findings.

### Antitumor efficacy of hNVs@Flu-EGCG in a subcutaneous tumor model

**Fig. 5:**
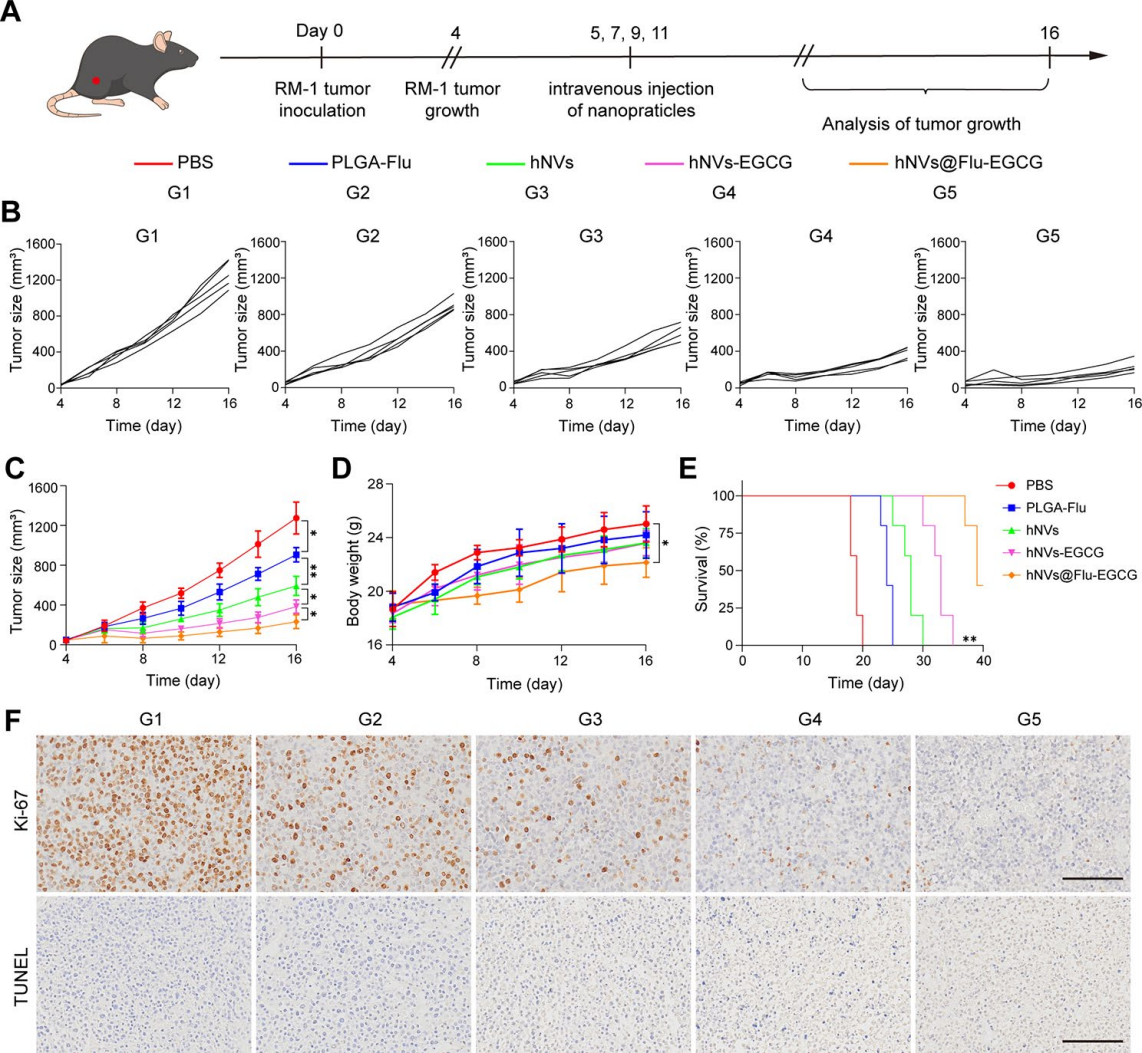
Antitumor effects of hNVs@Flu-EGCGin vivo. (**A**) Diagram illustrating the timetable of therapy following the C57BL/6 mouse RM-1 prostate tumor model. (**B**) Individual and (**C**) mean tumor growth patterns among varied groups (*n* = 5). (**D**) The body weight of mice in different groups (*n* = 5). (**E**) Survival plots of RM-1 tumor-carrying mice subjected to different therapies as noted. (**F**) Exemplary pictures exhibiting Ki-67 and TUNEL labeling of tumor sections carried out through immunohistochemistry. Scale bar, 100 μm. All data are expressed as mean ± S.D. NS: no significance, * *P* < 0.05, ** *P* < 0.01, *** *P* < 0.001, **** *P* < 0.0001

Encouraged by the excellent antitumor properties of hNVs@Flu-EGCG in vitro, we further assessed its antitumor capability based on the C57BL/6 mouse RM-1 prostate tumor model. To determine the antitumor activity of these nanoparticles, PBS, PLGA-Flu, hNVs, hNVs-EGCG and hNVs@Flu-EGCG were intravenously injected into C57BL/6 mice with subcutaneous RM-1 tumors once every other day for five times (Fig. 5A), and the size of the tumor and the mice’s body weight were monitored every other day. The growth rate of tumors of the PBS group was especially fast among the five groups. Distinctly, compared with other groups of treatments, administration of hNVs@Flu-EGCG to RM-1 tumor-bearing mice resulted in the lowest tumor volume (Fig. 5B and C). The tumor suppressing effect of hNVs group was better than PLGA-Flu group, which may be due to the strong anti-tumor immunity induced by membrane vesicles, and the reason that hNVs-EGCG group had better tumor suppressing effect than hNVs group could be the result of good anti-tumor ability of EGCG. As we can see in the Figure S10 (Supporting Information), The tumor volume was the smallest in hNVs@Flu-EGCG group, by reason of the remarkable tumor targeting capability and exceptional combined therapeutic effect of these nanoparticles. At the same time, a decrease in body weight was observed in the treatment groups, which could be attributed to the faster tumor growth rate in the PBS group compared to other groups (Fig. 5D). Remarkably, the overall survival of tumor-bearing mice had been significantly prolonged under the treatments of nanoparticles, among which the hNVs@Flu-EGCG group achieved the most terrific effect (Fig. 5E). Ki-67 immunohistochemistry and TdT-mediated dUTP Nick-End Labeling (TUNEL) staining further confirmed the effective inhibition of tumor cell proliferation. As is shown in Fig. 5F, tumor samples in treatment groups demonstrated reduced cell proliferation (Ki-67) and induced apoptosis (TUNEL), with the hNVs@Flu-EGCG group exhibiting the best therapeutic effect.

### Antitumor immune response of hNVs@Flu-EGCG in a subcutaneous tumor model

**Fig. 6:**
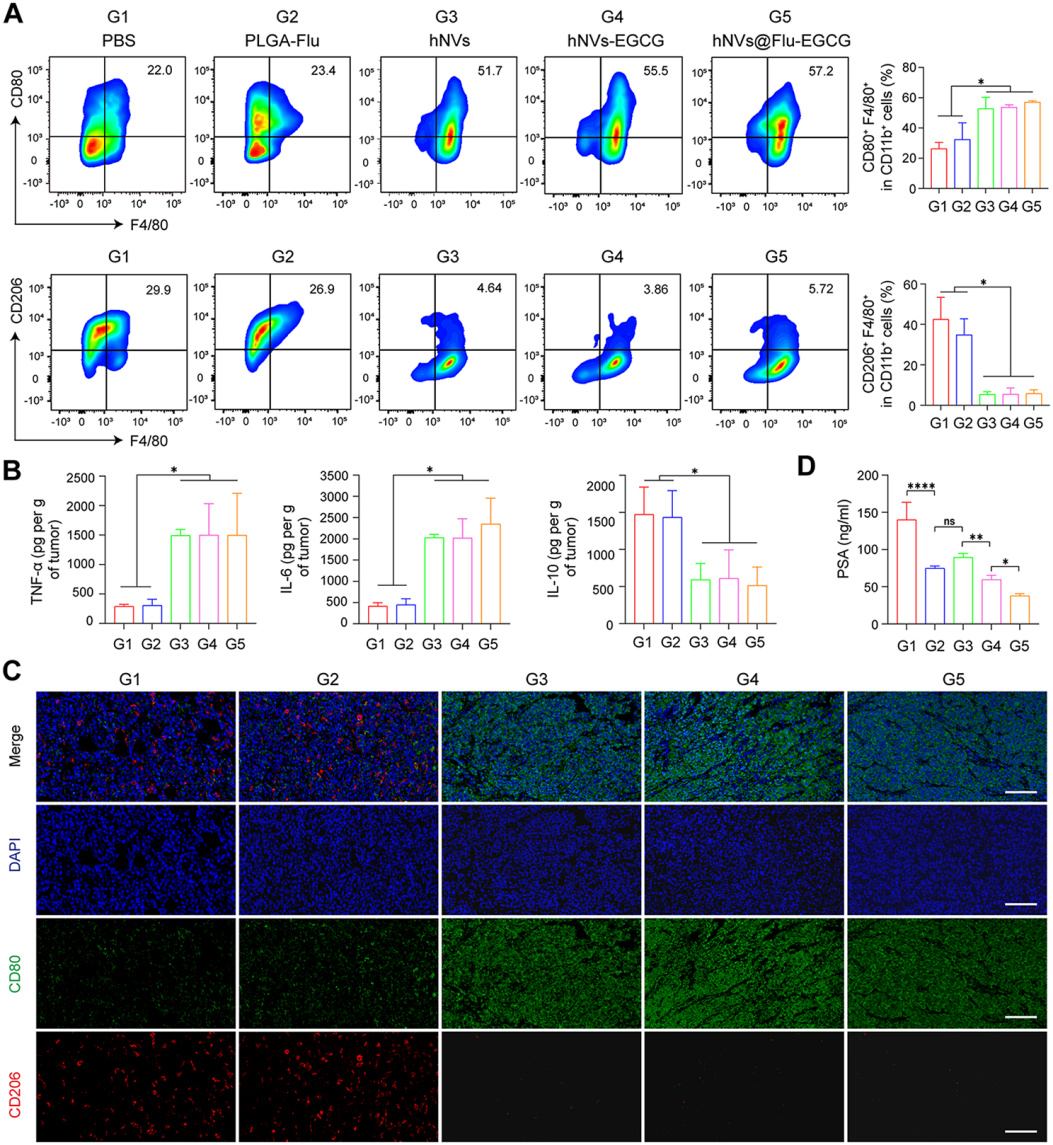
The hNVs@Flu-EGCG activated the immune system in vivo. (**A**) Images of flow cytometric analysis showing M1-like macrophages (CD80^+^) and M2-like macrophages (CD206^+^) gating on F4/80^+^CD11b^+^CD45^+^ cells within the tumor microenvironment following different therapies (*n* = 3). (**B**) Evaluation of levels of TNF-α, IL-6, and IL-10 cytokines in murine tumors post various treatments (*n* = 3). (**C**) Immunofluorescence images demonstrating infiltration of macrophages in tumors among diverse experimental cohorts. Scale bar, 100 μm. (**D**) Evaluation of serum PSA expression in RM-1 tumor-bearing mice following diverse treatments (*n* = 5). All data are expressed as mean ± S.D. NS: no significance, * *P* < 0.05, ** *P* < 0.01, *** *P* < 0.001, **** *P* < 0.0001

After verifying the treatment effectiveness of these nanoparticles, we next evaluated the mechanisms of immune responses in vivo by flow cytometry. Due to the introduction of vesicles, superior anti-tumor immunity has been triggered. We found that hNVs, hNVs-EGCG and hNVs@Flu-EGCG groups had a high proportion of M1 macrophages (CD80^+^) than PBS and PLGA-Flu groups, and M2 macrophages (CD206^+^) were lower in hNVs, hNVs-EGCG and hNVs@Flu-EGCG groups than those in PBS and PLGA-Flu groups, which indicated the enhanced tumor-suppressing effects (Fig. 6A). M1 macrophages primarily demonstrate anti-tumor effects through the secretion of cytokines like tumor necrosis factor-α (TNF-α) and interleukin-6 (IL-6), whereas M2 macrophages primarily release anti-inflammatory cytokines such as interleukin-10 (IL-10), which have promoting effects on tumor growth. Subsequently, the ELISA results of the tumor tissue demonstrated that the higher levels of TNF-α and IL-6 in hNVs, hNVs-EGCG and hNVs@Flu-EGCG groups and lower levels of IL-10 in hNVs, hNVs-EGCG and hNVs@Flu-EGCG groups compared to PBS and PLGA-Flu groups, showed the same result as before (Fig. 6B). Furthermore, immunofluorescence staining of tumor tissues was also conducted to visually determine the infiltration of immune cells after different treatments. Compared with tumors treated with PBS and PLGA-Flu, the fluorescence signals of CD80^+^ macrophages were significantly enhanced and CD206^+^ macrophages decreased in tumors treated with hNVs, hNVs-EGCG and hNVs@Flu-EGCG (Fig. 6C). Meanwhile, PSA is an important serological marker for prostate cancer, and the reduction of PSA content foreshadows the decrease of proliferation and increase of apoptosis in prostate cancer cells [35]. Flu and EGCG directly target prostate cancer cells, preventing the production of serum PSA, inducing apoptosis, and ultimately inhibiting tumor growth (Fig. 6D).

## Discussion

In summary, we developed the novel nanoparticles, hNVs@Flu-EGCG, which showed great promise for the combination therapy in prostate cancer. Specifically, hNVs were originated from EcN and RM-1 cell membrane, exhibited the ability to prolong the circulation time of drugs in the bloodstream and significantly improve the accumulation at tumor sites. PLGA-Flu acted on androgen receptors, reducing PSA level, inducing apoptosis and inhibiting the proliferation of prostate cancer cells. Importantly, hNVs facilitated the conversion of TAMs from the M2 phenotype to the M1 phenotype, which has thus confirmed their potential in inhibiting tumor growth. At the same time, EGCG, used as an effective anti-tumor agent, suppressed cancer cell proliferation and promoted apoptosis by inhibiting the NF-κB signaling pathway. Thus, hNVs and EGCG block the two major mechanisms of castration resistance, enhance the effectiveness of combination therapy. The key advantage of combination therapy is its ability to target multiple pathways involved in the growth and advancement of prostate cancer. By utilizing different treatment modalities that act on distinct biological processes, combination therapy could effectively disrupt the cancer’s ability to adapt and resist individual treatments. This could enhance treatment efficacy, overcome treatment resistance, and ultimately improve the prognosis for patients with prostate cancer.

The main constraints of ADT are primarily related to the activation of the NF-κB signaling pathway and the high infiltration level of M2-like macrophages in the TME [36]. Prostate cancer is a prevalent malignant tumor in the male genitourinary system, with its development and advancement being intricately linked to androgen metabolism [37]. ADT is the efficacious and quintessential treatment, working by lowering the androgen levels within the body. This is crucial as androgens have the ability to stimulate the growth of prostate cancer cells [38]. However, systemic administration of androgen receptor antagonists can cause notable toxic side effects on normal tissues, and aberrant activation of NF-κB signaling pathway can lead to drug resistance [39]. EGCG has been proven to have significant therapeutic advantages for prostate cancer in multiple clinical trials. Research shows that EGCG may effectively suppress the proliferation and metastasis of prostate cancer cells, induce apoptosis, and inhibit the production of PSA, thereby effectively preventing tumor growth and spread [40]. Through the inhibition of the NF-κB signaling pathway, EGCG has the potential to reverse prostate cancer resistance to ADT, decrease NF-κB protein expression, and halt tumor progression.

Cell-free strategies, such as CVs, are gaining traction in the field of disease treatment [41]. Due to their unique attributes of specificity, stability, and compatibility with the human body, EVs have garnered significant attention as potential carriers for drug delivery. This has led to in-depth research on their ability to endow cargo loaded within with enhanced biological interface. The introduction of natural membrane substrates on the surface of nanoparticles has emerged as an attractive platform, gaining more applications beyond traditional nanomedicine fields [42]. CVs-camouflaged nanoparticles mimic the characteristics of the source cells, combining the advantages of natural and artificial nanomaterials, and providing a wide range of functions, such as high level of safety, natural long circulation, good immune evasion properties, and tumor targetability [43]. TAMs highly infiltrate most solid tumors and are among the most abundant immune cells in the TME, and M2-like TAMs inhibit immunity and support tumor growth, invasion, and angiogenesis, while M1-like TAMs are characterized as anti-tumor phenotypes. The reprogramming of M2-like TAMs into M1-like TAMs has emerged as an efficient strategy for immunotherapy [44]. EcN promote the transition of M2-like TAMs into M1-like TAMs, and EcN-derived OMVs serve as the nanocarriers to deliver drugs into tumor tissues and have the ability to reprogram M2 into M1-like macrophages.

In future clinical practice, the application of cell membrane vesicles-derived nanoparticles may open up exciting new opportunities in the field of personalized precision medicine. Novel nanoparticles have a favorable safety profile, low toxicity and cost, high stability and efficiency of modification. Given the current treatment status of prostate cancer and the particularity of prostate cancer, more attention should be paid to the application of combination therapy, continuously exploring more effective combination treatment regimens with minimal side effects to bring better treatment outcomes and quality of life to prostate cancer patients [45]. Compared with traditional drugs, hNVs@Flu-EGCG show promising potential as the drug delivery vehicle for the treatment of prostate cancer, conspicuously improve the drug loading efficiency. Meanwhile, side effects associated with conventional non-targeted therapies can be avoided after systemic administration, and ADT resistance caused by activation of NF-κB pathway and M2 macrophages infiltration can also be eschewed. Thus, for prostate cancer patients, combination therapy has obvious advantages. In spite of supplemental studies being requisite to further fathom, we conceive hNVs@Flu-EGCG furnish a firm foundation for continuous development of novel nanoparticles, which have tremendous potential in the future biomedical application.

### Electronic supplementary material

Below is the link to the electronic supplementary material.

Supplementary Material 1

## Data Availability

No datasets were generated or analysed during the current study.
