# Supplementary material for: Tea polyphenol-engineered hybrid cellular nanovesicles for cancer immunotherapy and androgen deprivation therapy
Source: J Nanobiotechnology. 2024 Apr 18;22:192. doi: 10.1186/s12951-024-02458-9 (PMC11025249; doi:10.1186/s12951-024-02458-9)
Supplement: Supplementary file 1 — Supplementary Material 1 [file 12951_2024_2458_MOESM1_ESM.pdf]

Supporting Information

Tea Polyphenol-Engineered Cellular Nanovesicles for Cancer Immunotherapy and Androgen Deprivation Therapy

Yiming Guo[†,‡], Jicheng Wu[‡,◊], Lefan Chen[†], Lujie Liu[‡], Tianxiang Bi[†], Yuanwei Pan[‡], Qian-Fang Meng[‡], Chaoliang Wang[†], Lang Rao[‡,*], and Qi Li[†,*]

[†] Department of Urology, The First Affiliated Hospital of Zhengzhou University, Zhengzhou 450052, China.

[‡] Institute of Biomedical Health Technology and Engineering, Shenzhen Bay Laboratory, Shenzhen 518132, China.

[◊] Cancer Center, Renmin Hospital of Wuhan University, Wuhan 430060, China.

* Corresponding e-mail: richee@zzu.edu.cn (Q.L.); lrao@szbl.ac.cn (L.R.).

Supplementary Figures

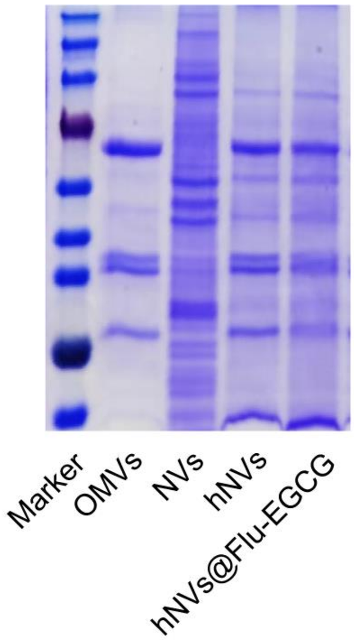

Fig. S1. SDS-PAGE analysis of OMVs, NVs, hNVs, and hNVs@Flu-EGCG.

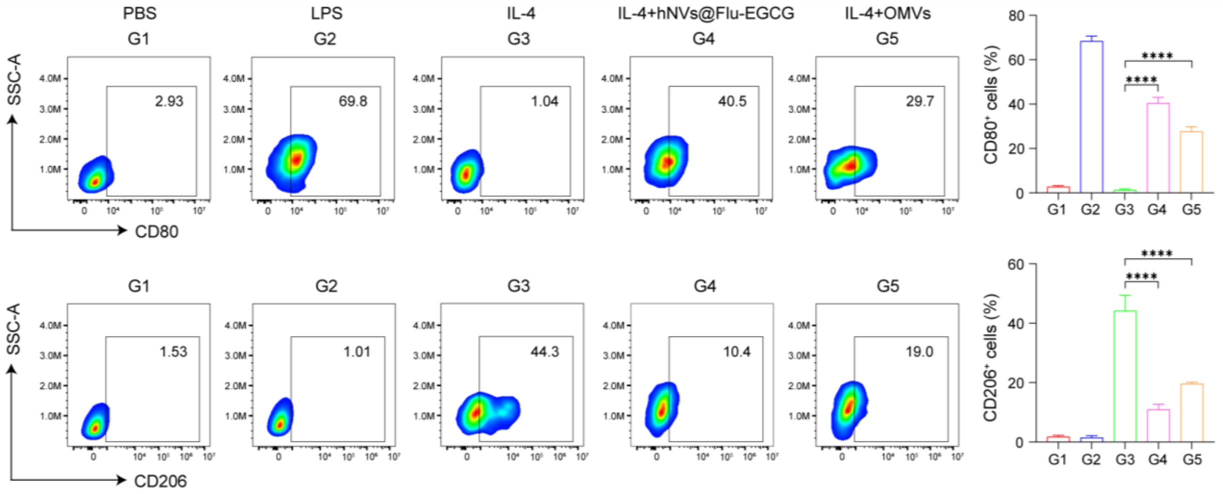

Fig. S2. The reprogramming capabilities of OMVs and hNVs@Flu-EGCG were analyzed by flow cytometry (n = 3).

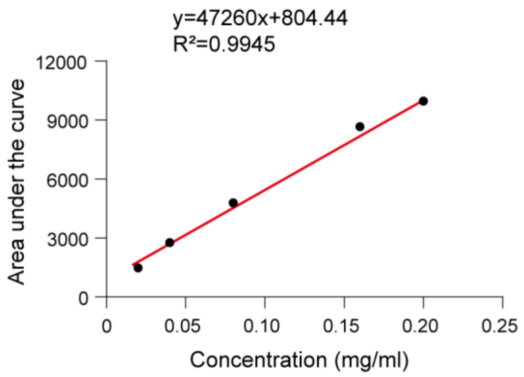

Fig. S3. The area under the curve of the chromatograms of free Flu was plotted against concentration to make a standard curve by HPLC.

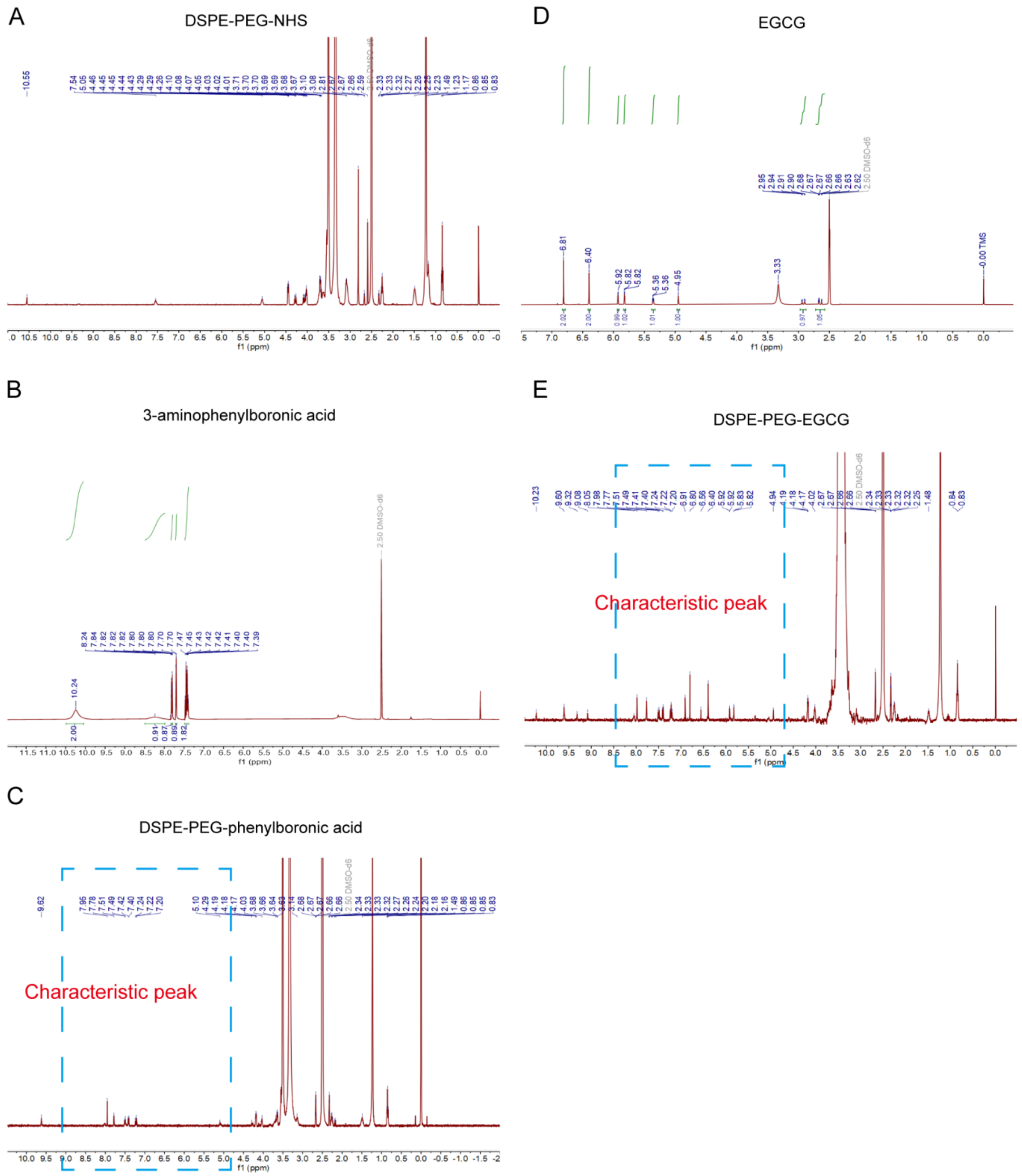

Fig. S4. The chemical components were characterized by Nuclear Magnetic Resonance (1H NMR) spectrum. (A) DSPE-PEG-NHS, (B) 3-aminopheylboronic acid, (C) DSPE-PEG-phenylboronic acid, (D) EGCG, and (E) DSPE-PEG-EGCG.

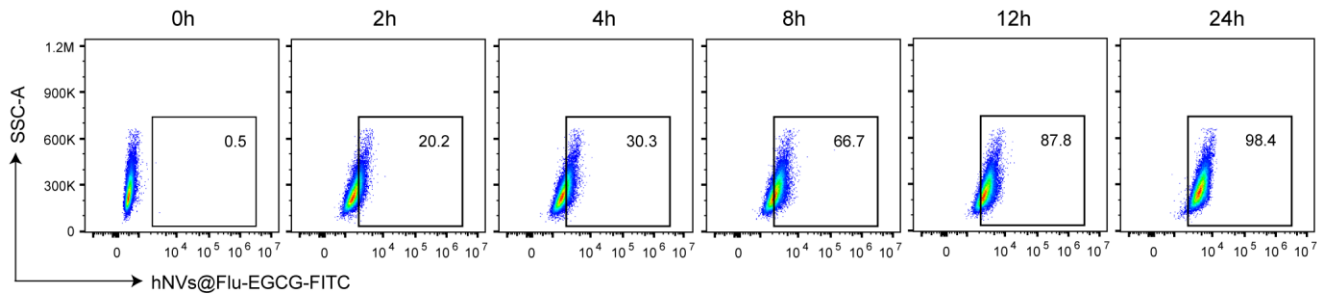

Fig. S5. Representative flow cytometric analysis images of the uptake ratio of hNVs@Flu-EGCG in RM-1 cells (n = 3).

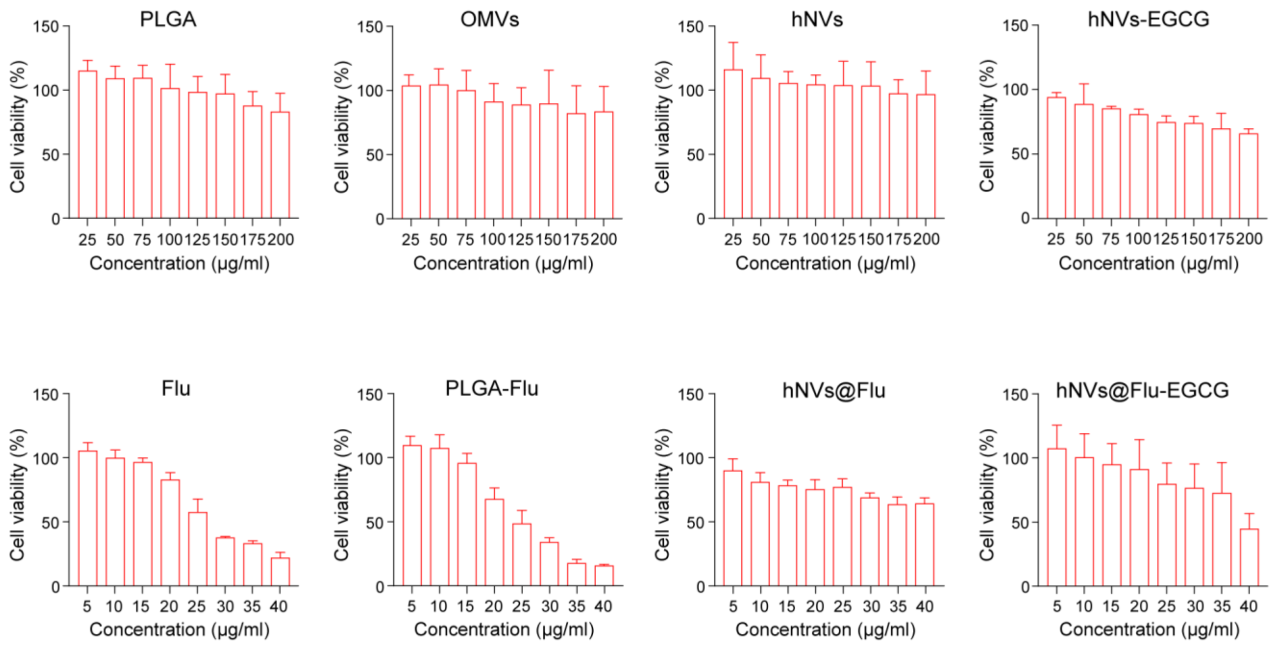

Fig. S6. CCK-8 results of RM-1 cells cultured with different components (n = 5).

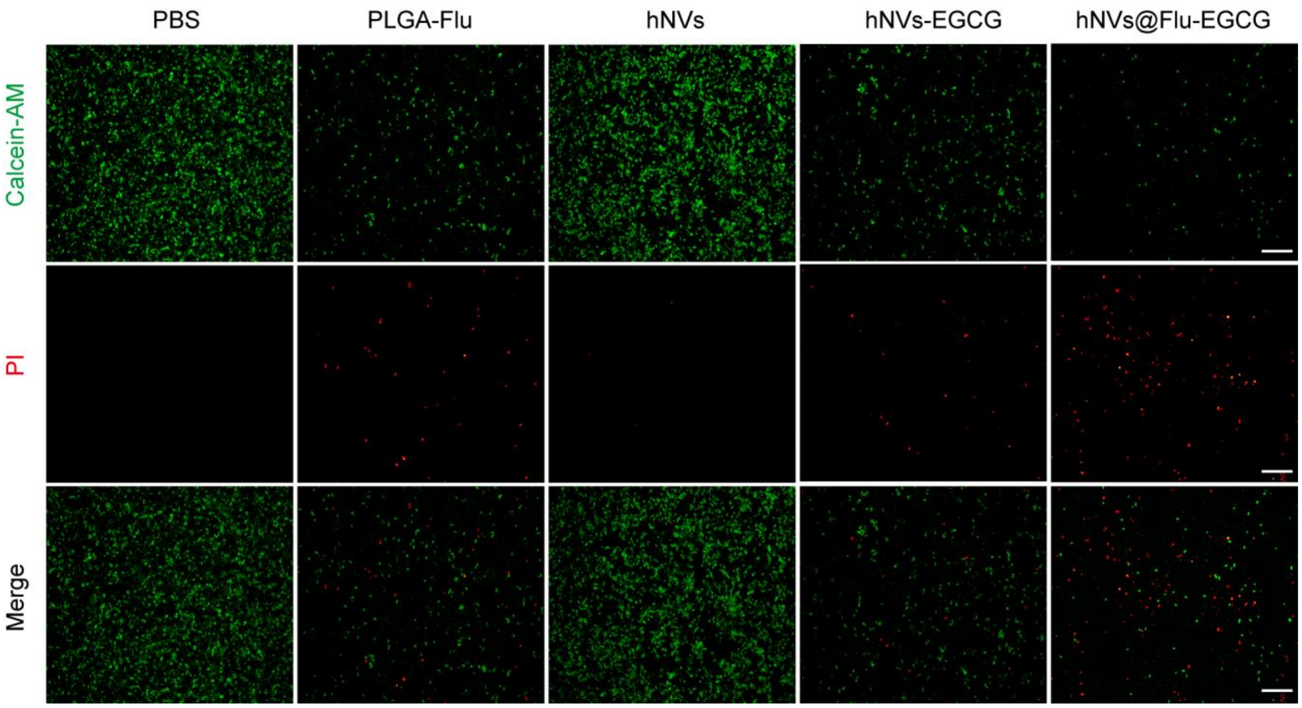

Fig. S7. The images of RM-1 cells costained with calcein-AM (green fluorescence) and PI (red fluorescence) after various treatments. Scale bar, 50 μm.

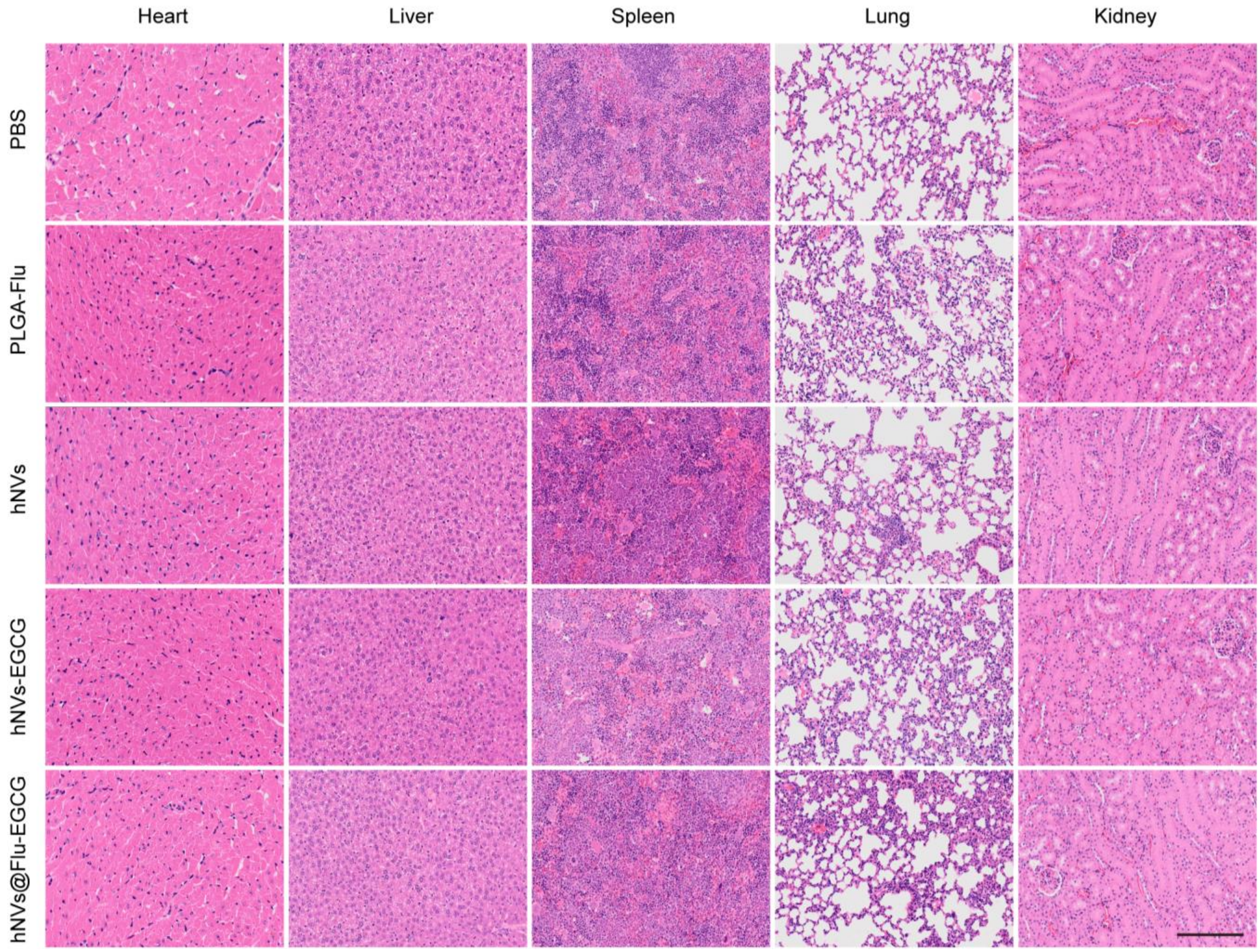

Fig. S8. Representative images of mice organ sections stained with H&E in different groups. Scale bar, 100 μm. All data are expressed as mean ± S.D. (n = 5).

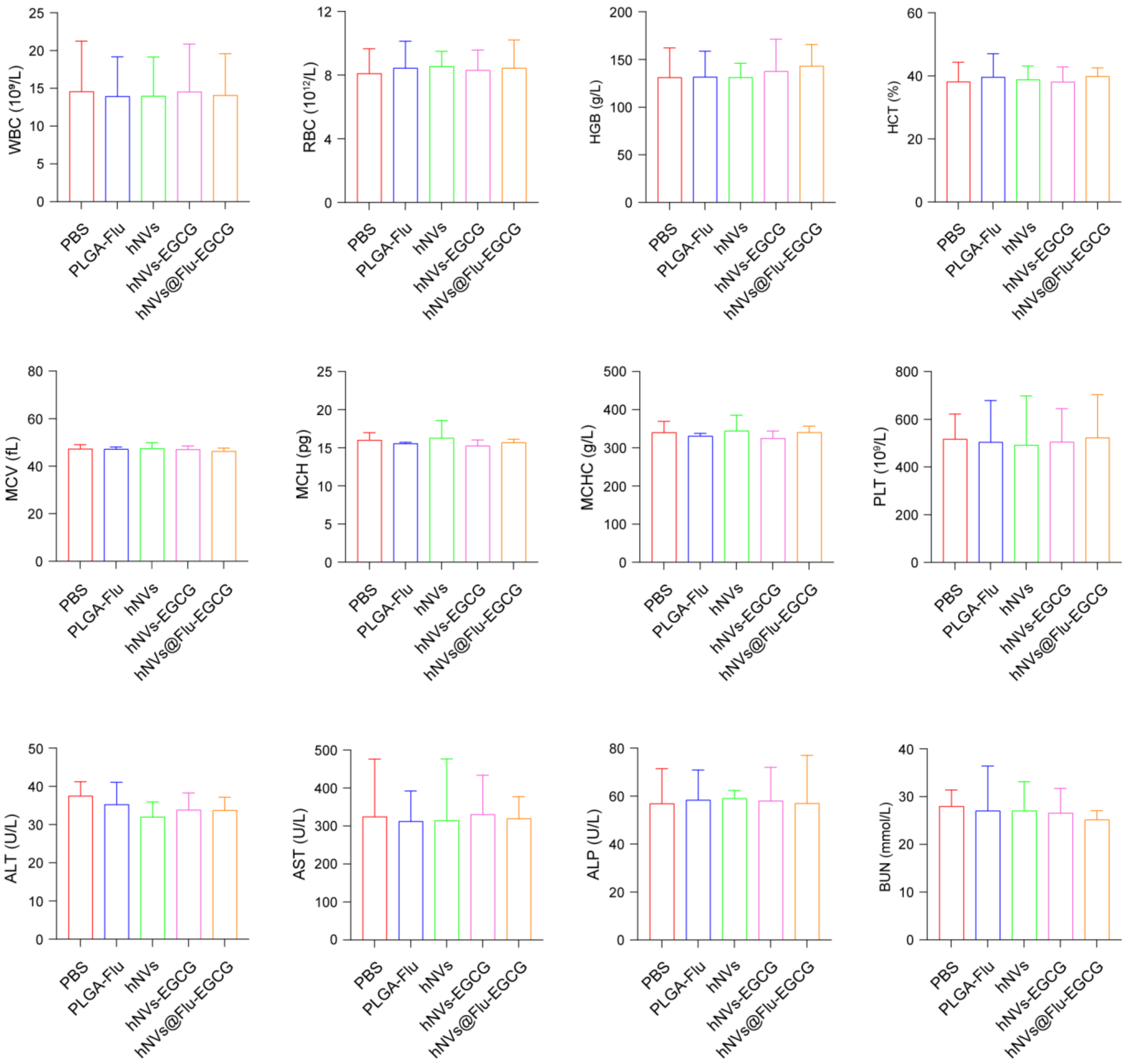

Fig. S9. The results of blood routine and blood biochemistry. WBC: white blood cell, RBC: red blood cell, HGB: hemoglobin, HCT: hematocrit, MCV: mean corpuscular volume, MCH: mean corpuscular hemoglobin, MCHC: mean corpuscular hemoglobin concentration, PLT: platelets, ALT: alanine transaminase, AST: aspartate aminotransferase, ALP: alkaline phosphatase, BUN: blood urea nitrogen.

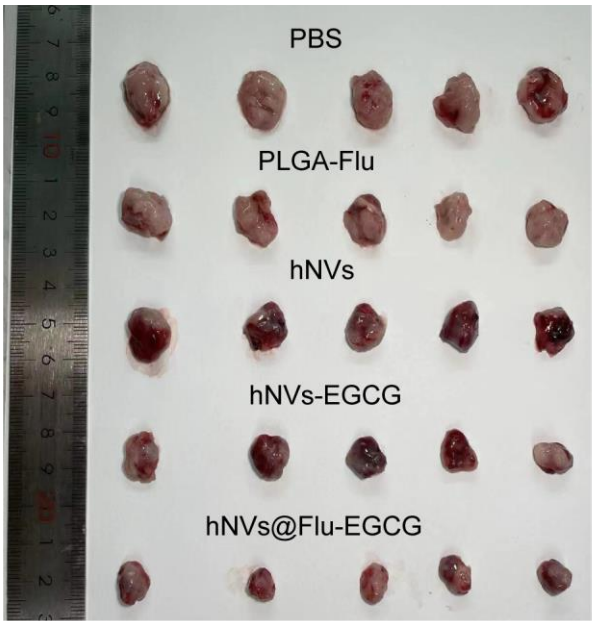

Fig. S10. Photographs of tumor in different groups after treatment (n = 5).

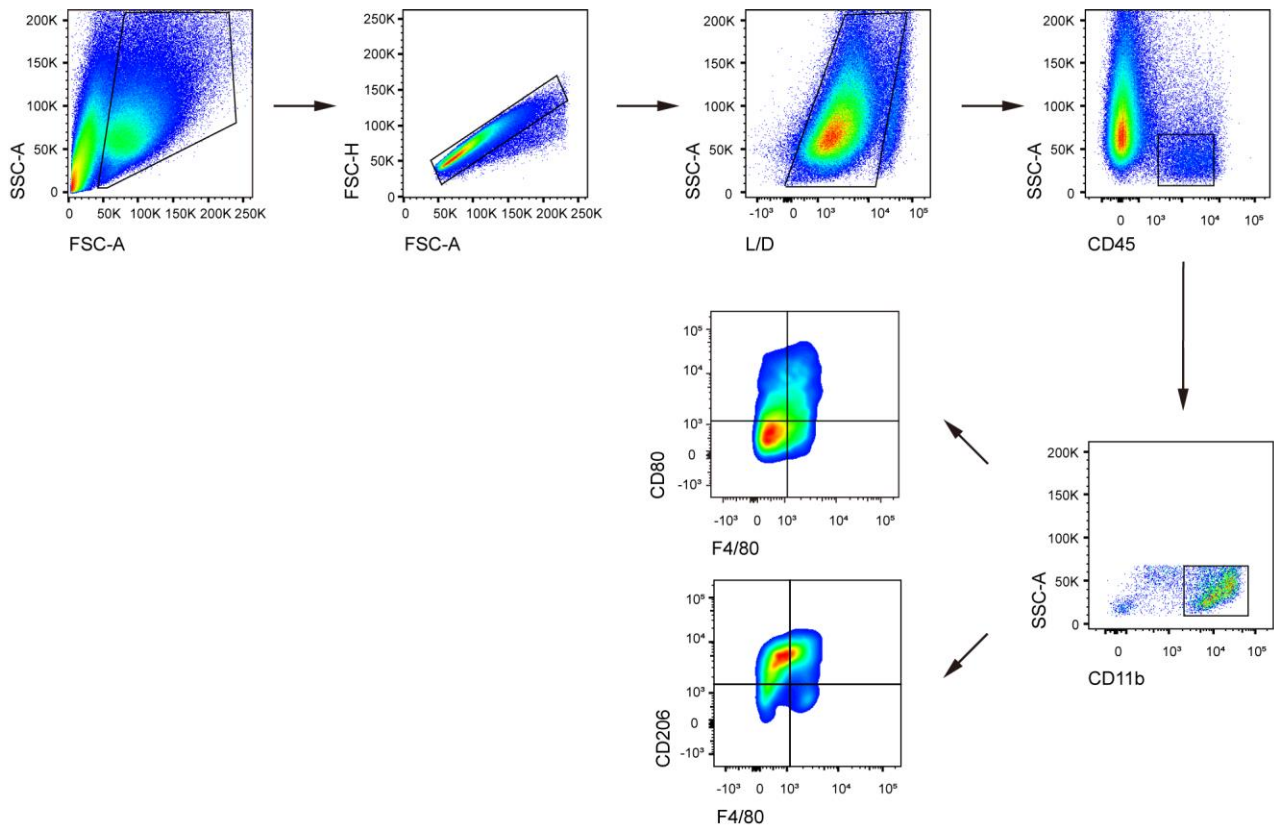

Fig. S11. Graphically account for flow cytometry gating strategy to sort $CD206^+F4/80^+$ M2-like and $CD80^+F4/80^+$ M1-like macrophages in tumor tissues gating on $CD11b^+CD45^+$ cells presented on Figure 6A.
